# Nano-Metal Hydrogen Therapy Targeting Immune Cells Drives Synergistic Anti-Tumor Effects

**DOI:** 10.3390/molecules31173058

**Published:** 2026-08-31

**Authors:** Yuxuan Ding, Min Ma, Jianing Wang, Zixin Zhang, Jingyu Wu, Linxiang Zhou, Xiaoyan Cao, Libing Liu

**Affiliations:** 1 Department of Nutrition and Health, China Agricultural University, Beijing 100193, China; dingyxuan2024@163.com (Y.D.); mamin_st@163.com (M.M.); jianing20010726@126.com (J.W.); btbtwbnj1@163.com (Z.Z.); wujingyu1006@163.com (J.W.); linxiang797@163.com (L.Z.); caoxiaoyan93314@163.com (X.C.); 2 Key Laboratory of Precision Nutrition and Food Quality, Beijing 100193, China; 3 Beijing Advanced Innovation Center for Food Nutrition and Human Health, Beijing 100193, China

**Keywords:** hydrogen immunotherapy, hydrogen therapy, nanometals, immune modulation, tumor

## Abstract

Hydrogen is employed as a therapeutic gas in the treatment of stroke, tissue repair, and cancer, owing to its excellent biocompatibility, antioxidant properties, anti-inflammatory effects, and regulation of cellular metabolism. Moreover, nanometallic materials generate hydrogen through pH-responsive, light-responsive, ultrasound-responsive, and electrical stimulation and play a pivotal role in cancer therapy by activating anti-tumor immune responses, reversing immunosuppressive microenvironments, inducing immunogenic cell death, and sensitising radiotherapy and chemotherapy. Consequently, hydrogen therapy based on nanometallic materials has emerged as a novel research focus in cancer treatment. This review systematically elucidates the unique anti-cancer immunobiological effects of hydrogen therapy based on novel nanometallic materials. It meticulously analyses the reversal effects of different metals (Ca, Mg, Fe, Cu, Yb, etc.) in overcoming obstacles within the cancer immune cycle (including antigen presentation, T-cell activation, and resistance mechanisms). It highlights the structure–activity relationships between ‘metal type-specific activity-immune effects’ in the latest hydrogen therapies, elucidates the primary signaling pathways involved in hydrogen-mediated immune regulation, and systematically summarises breakthrough advances in how hydrogen therapy modulates immune responses against tumors. Building upon current cancer treatment trends, this review will synthesise key factors from clinical translation and immunological research perspectives to propose future directions for the field, addressing prevailing challenges.

## 1. Introduction

Disease represents one of the most severe challenges to human survival, development, and well-being [1]. Over recent decades, cancer treatment paradigms have progressively evolved from ‘surgical resection plus radiotherapy/chemotherapy for prognosis’ strategies towards immunotherapies exemplified by immune checkpoint inhibitors (ICIs) [2] and chimeric antigen receptor T-cell (CAR-T) [3] therapies. Immunotherapy activates the patient’s own immune system to combat cancer, enabling specific killing and long-term treatment that progressively eradicates systemic lesions or suppresses tumor spread. However, core obstacles such as immune escape, difficulties in T-cell infiltration, and tumor antigen heterogeneity severely limit the therapeutic efficacy of immunotherapies centred on immune checkpoint inhibitors, adoptive cell therapies, and cancer vaccines [4]. Consequently, there is an urgent clinical need to develop novel therapies that reduce treatment toxicity, overcome therapeutic resistance, improve the tumor microenvironment (TME), and activate immune responses in cancer treatment.

Gas-assisted therapy and diagnostics, as an emerging field, may serve as therapeutic or adjunctive treatment molecules for diseases due to their eco-friendly nature and minimal side effects [5], such as methane (CH_4_) [6], nitric oxide (NO) [7], carbon monoxide (CO) [8], hydrogen sulfide (H_2_S) [9], and hydrogen gas (H_2_) [10]. Owing to its small molecular weight and electrically neutral nature, H_2_ readily permeates biological membranes, diffusing into the nucleus and mitochondria. It specifically neutralizes reactive oxygen species such as hydroxyl radicals and peroxynitrite, thereby reducing oxidative damage and maintaining the body’s redox balance [11,12]. Concurrently, H_2_ neutralizes the acidic tumor microenvironment, disrupting TME equilibrium, whilst remaining unaffected by signaling molecules such as hydrogen peroxide and superoxide anion. This enables synergistic inhibition of mitochondrial respiration in cancer cells [13]. It is precisely the dual nature of H_2_ that allows it to regulate immune responses and kill cancer cells through ROS accumulation while protecting normal cells, ultimately achieving low toxicity and high sensitivity in anticancer therapy.

**Scheme 1:**
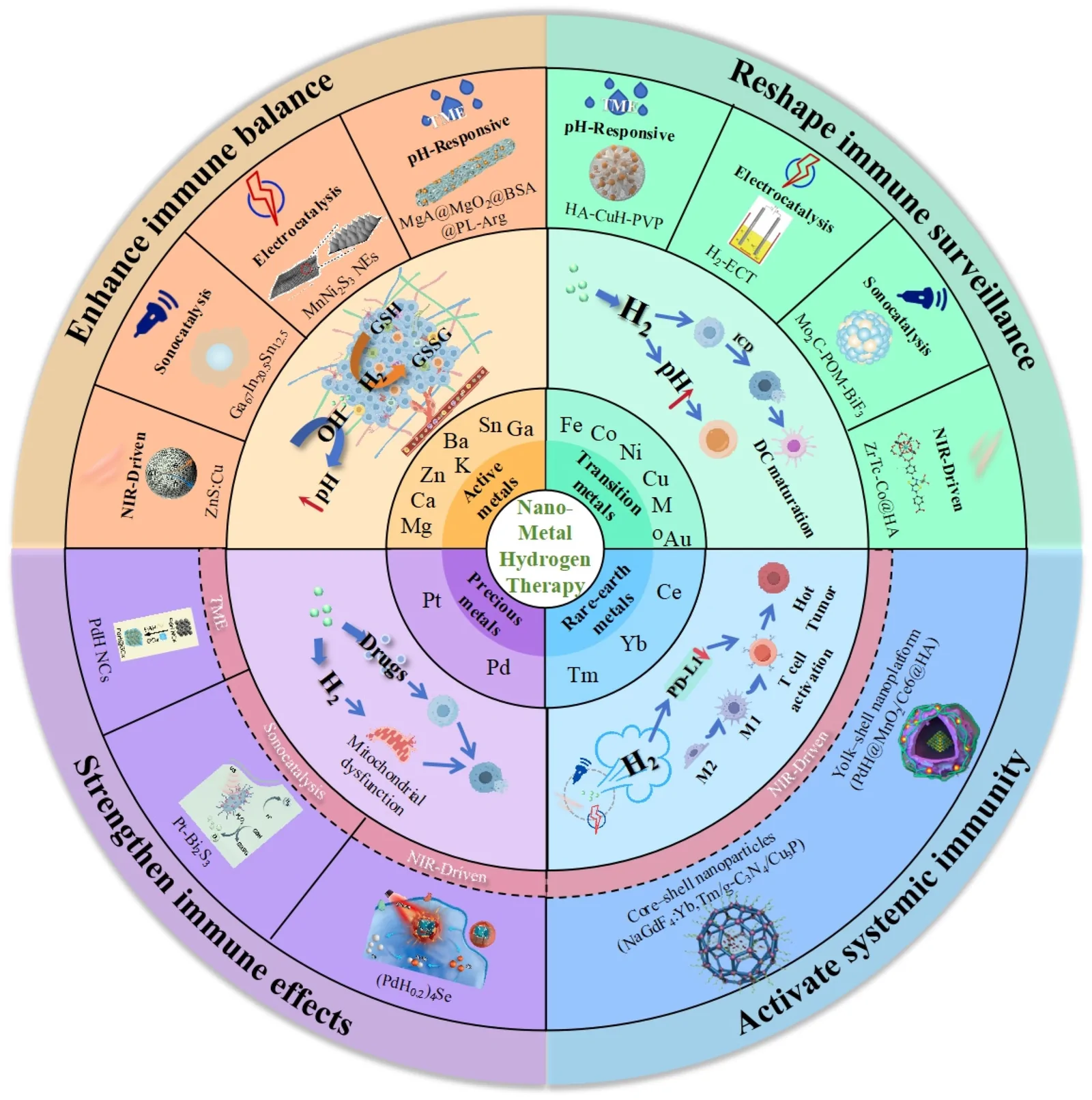
Schematic diagram of activation conditions and immunoregulatory mechanisms for nanometallic hydrogen immunotherapy. Nano-metallic materials comprise composite substances primarily composed of precious metals, transition metals, reactive metals, and rare earth metals with hydrogen-producing properties. These materials release H_2_ and corresponding metal ions through pH-responsive, light-responsive, electrically stimulated, and acoustically stimulated mechanisms. This enables hydrogen-mediated regulation of immune responses, including reversing tumor-suppressive microenvironments, remodelling immune surveillance, and synergising immune effects. This achieves the transformation of “cold” tumors into “hot” tumors, thereby enabling more sustained and effective tumor suppression or treatment.

Engineered H_2_ production strategies based on nanometallic materials can achieve spatiotemporal precision control of H_2_ through stimulus-responsive mechanisms, thereby activating or enhancing immune responses within the cancer immunological cycle to synergistically combat tumors. Notably, hydrogen’s immunomodulatory capacity manifests primarily through reversing immunosuppression, remodelling immune surveillance, and synergising immune effects [14]. On the one hand, H_2_ protects immune cells from oxidative stress damage and promotes macrophage polarisation towards the M1 anti-tumor phenotype, thereby reversing the tumor-suppressive microenvironment [15]. On the other hand, H_2_ induces reactive oxygen species (ROS) bursts within tumor cells, promoting immunogenic cell death (ICD) and releasing damage-associated molecules. This activates dendritic cell maturation and T-cell infiltration [16]. Consequently, radiotherapy/chemotherapy agents can be integrated into nanometallic materials, leveraging H_2_ to reverse tumor immunosuppressive microenvironments. This enhances tumor sensitivity to drugs, enabling synergistic therapy [17]. With the advantages of targeted sustained-release and hydrogen-mediated immune modulation offered by nanometal materials, hydrogen therapy based on these materials holds significant potential in cancer treatment (Scheme 1).

Most existing reviews have focused on material design and the biological effects of hydrogen, but they lack a systematic discussion of the physicochemical properties of hydrogen-producing nanomaterials and their regulatory effects from the perspective of tumor immune regulation. Therefore, this review focuses on the interdisciplinary connections between nanoscience and tumor immunology, moving beyond the traditional classification framework centered on nanomaterial development and delivery, with the aim of elucidating the unique effects of nanomaterial-based hydrogen therapy in regulating the tumor immune cycle. It innovatively proposes a framework centered on immune regulatory mechanisms for summarizing and elaborating on hydrogen therapy. We provide an overview of the “initiating signals” that stimulate H_2_ production in response to nanomaterials for cancer treatment, including pH, light, electricity, and magnetism, among others. Additionally, we have detailed the existing hydrogen therapy based on nanometal materials throughout the entire process of treating different tumors, covering the stages of material stimulation response, activation, and immune effects. The aim is to improve the effect of hydrogen therapy based on metal nanomaterials and supplement hydrogen-mediated cancer immunotherapy. Furthermore, we evaluate the clinical translation potential of metallic nanomaterials in recent years. Based on this critical assessment, we propose unique perspectives on the future development of immune effects and clinical translation for hydrogen therapy utilizing metallic nanomaterials in tumor treatment.

## 2. The “Trigger Signal” for Nano-Metal Hydrogen Therapy

Hydrogen therapy refers to a strategy that involves the inhalation of H_2_ or the intake or injection of hydrogen-releasing substances to establish a certain concentration of H_2_ enrichment in the body. This approach leverages the biological effects of H_2_, such as selective antioxidant, anti-inflammatory, and anti-apoptotic properties, to achieve therapeutic or adjunctive treatment goals for various diseases. In 2007, Ohsawa et al. [18] published a paper in Nature Medicine demonstrating that low concentrations of H_2_ (2%) could selectively neutralize highly toxic ROS and effectively alleviate acute cerebral ischemia–reperfusion injury in rats, thereby initiating over a decade of development in hydrogen therapy. Subsequently, numerous studies have confirmed that H_2_ possesses multiple functions, including selective antioxidant, anti-inflammatory, and anti-apoptotic effects. Due to its small molecular size, strong diffusivity, and high safety profile, H_2_ has shown excellent therapeutic potential in various disease models, such as Parkinson’s disease, inflammatory bowel disease, melanoma, and the toxic side effects of radiotherapy and chemotherapy in cancer. It can improve patient prognosis and quality of life, leading to the gradual clinical application of hydrogen therapy [19]. Moreover, hydrogen’s ability to regulate immune responses and enhance sensitivity to radiotherapy and chemotherapy has increasingly highlighted its advantages in the treatment of inflammatory diseases and cancer. However, traditional methods of hydrogen administration (hydrogen inhalation, hydrogen-rich water, and hydrogen-rich saline injection) struggle to effectively maintain the desired concentration and therapeutic range of H_2_, limiting its widespread clinical application. Addressing challenges such as the low efficiency of H_2_ delivery in the body, poor targeting of lesions, difficulty in sustaining effective concentrations, and understanding its mechanisms of action has become an urgent issue in the development of hydrogen medicine [20]. Therefore, the development of intelligent and controllable nanomaterials (such as hydrogen-producing nanometal materials and hydrogen-producing metal–organic frameworks) has emerged as a promising direction for advancing hydrogen therapy.

### 2.1. TME-Responsive Metallic Nano-Materials

**Figure 1:**
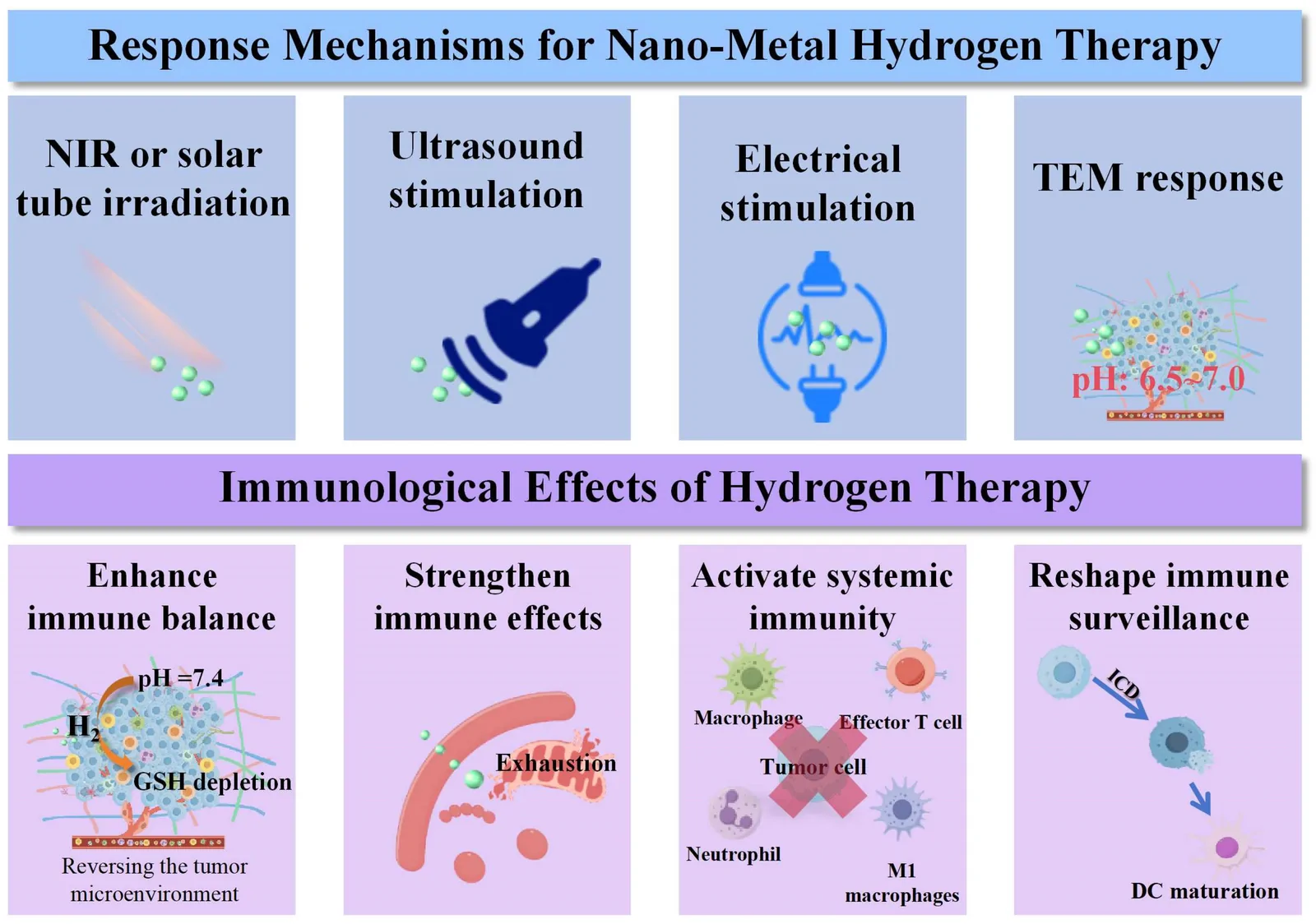
Schematic Diagram of Different Response Mechanisms of Hydrogen-Producing Metal Nano-materials and Methods for Regulating Hydrogen Immunity. Metal nanomaterials respond to different environments by releasing substances such as metal ions and H_2_. These substances work synergistically to influence the homeostasis of the tumor microenvironment and activate or enhance immune responses, thereby transforming “cold” tumors into “hot” tumors and enabling sustained treatment.

In material design, TME involves factors such as hypoxia, high glutathione (GSH) levels, low pH (acidic environment), ATP overexpression and enzymes, making TME the most commonly utilized “trigger signal”. Firstly, in oxygen-rich environments, cancer cells rely on a glycolytic metabolic pathway (termed the Warburg effect), thereby producing large quantities of acidic by-products. This results in most tumor tissues exhibiting an acidic (pH = 6.0~7.0, termed acidosis) and hypoxic environment. The acidic environment inhibits ERK activity in the cytoplasm, enhances mitochondrial respiratory function, and thereby reshapes the metabolic fate of cancer cells, initiating an energy metabolism pathway that suppresses glycolysis dependence and activates mitochondrial respiration [21] (Figure 1). Based on the core concept that “tumor pH determines the energy metabolism balance of cancer cells”, the design of acid-responsive hydrogen-producing metal nanomaterials focuses on the regulation of electronic structures. By integrating recognition units (acid-responsive) and functional units (pH regulation and H_2_ generation), a system capable of dynamic response and controlled release is constructed. The primary metals in such materials are typically Mg, Ca, Cu, Zn, Pd, and Pt. To ensure stable delivery to tumor tissues and achieve controlled H_2_ release, inert shells such as SiO_2_ and hydrophilic/hydrophobic polymers like chitosan are strategically introduced onto the material surface [22]. These acid-responsive metal nanomaterials can not only hydrolyze to produce H_2_ as active centers but also utilize released metal ions (such as Mg^2+^ and Cu^2+^) and OH^−^ to reverse the acidic environment, thereby activating the cGAS-STING signaling pathway and modulating innate immunity [23].

Secondly, in tumor tissues, metabolic dysregulation and redox imbalance lead to the abnormal accumulation of ROS and reactive nitrogen species (RNS), forcing the upregulation of the antioxidant system in tumor cells to counteract oxidative stress, resulting in a significant increase in intracellular GSH concentrations. Concurrently, GSH is oxidized by oxidants to form GSSG, which can then be reduced and regenerated by glutathione reductase (GSR) using NADPH [24]. This process maintains a unique redox homeostasis within the tumor and has become a key factor driving tumor drug resistance. To respond to the high intracellular GSH environment, materials are often designed to incorporate Cu, Mn, disulfide bonds, and electron–hole pairs to enhance their redox sensitivity. Simultaneously, high GSH levels can reduce metal nanomaterials to produce H_2_, while the released metal ions (such as Mn^2+^ and Cu^2+^) can regulate the expression of antioxidant enzymes, leading to GSH depletion and influencing the expression of genes such as NF-κB and TNF, as well as the polarization of tumor-associated macrophages (TAMs), thereby amplifying the therapeutic effect against tumors.

Finally, under environmental stresses such as hypoxia and acidosis, tumor cells produce ATP intracellularly and release it into the extracellular space, leading to an abnormal accumulation and elevation of extracellular ATP concentrations [25,26]. However, extracellular ATP acts as a “double agent” that masks tumor immune evasion. On the one hand, it rapidly metabolizes ATP into adenosine via key enzymes such as CD39. Adenosine then binds to A2A or A2B receptors on immune cells like T cells and NK cells, utilizing the cAMP/PKA/CREB signaling pathway to inhibit T cell proliferation and activation, and promoting the expansion of immunosuppressive cells such as M2 macrophages, thereby rendering the tumor “cold” [27]. On the other hand, ATP can directly interact with P2RX7 or P2RY2 receptors on immune cells, thereby inhibiting the function of effector T cells and establishing an immunosuppressive microenvironment [28]. Currently, the design of metal-based nanomaterials for H_2_ production driven by high-level ATP is still under exploration, primarily focusing on the development of nanoenzymes to catalyze ATP consumption [29], and the dynamic response of ATP-metal interactions is viewed as a promising strategy for breakthroughs [30].

### 2.2. Light-Responsive Metallic Nano-Materials

The development of tumors is a dynamic and disordered process. As tumor cells proliferate rapidly, the tumor forms a multi-layered structure (also known as spatial heterogeneity) with internal hypoxia inhibition, central disordered proliferation, and external immune rejection. This makes it difficult for chemotherapy and radiotherapy drugs to penetrate the dense extracellular matrix (ECM) and diffuse from blood vessels into the tumor interior [31]. Precisely because drugs struggle to penetrate deep into the tumor core to exert their cytotoxic effects, the advantages of the spatially precise control and deep tissue penetration of light-responsive metal nanomaterials are highlighted, thanks to the high tissue penetration ability and controllability of near-infrared (NIR) light in regions I and II [32]. Strategies such as alloying, suppression of electron–hole pairs [33], heterostructure construction [34], and photothermal cascade catalysis [35] have been employed to address the challenge that hydrogen-producing metallic materials face in responding to NIR stimulation due to limitations imposed by the “bandgap-potential” relationship. Compared to other stimulation-responsive methods, photoresponsive metallic nanomaterials often generate heat upon light stimulation, thereby inducing a burst of ROS within tumor cells. This promotes the release of damage-associated molecular patterns (DAMPs), triggers ICD, activates dendritic cell maturation and antigen presentation functions, and consequently stimulates adaptive immune responses [36]. Additionally, these materials have been shown to release H_2_ upon NIR triggering, which not only ablates the primary tumor but also induces a systemic antitumor immune memory response, thereby exerting an inhibitory effect on distant tumors [37,38]. This therapeutic strategy, which releases H_2_ directly into the tumor’s “core region”, more effectively suppresses tumor spread and recurrence. It also directly targets “immune-suppressive barriers” within the tumor microenvironment, such as TAMs and regulatory T cells (Tregs), dismantling immune suppression in the core region and facilitating the delivery and killing action of anticancer drugs [39].

### 2.3. Acoustic-Driven Metallic Nano-Materials

To overcome the limitations of tissue penetration in cancer treatment and the challenges of postoperative healing, and to achieve the goal of integrating diagnosis and treatment, sonodynamic therapy offers a new solution for cancer treatment due to its non-invasive nature, the deep tissue penetration capabilities of ultrasound, and its mechanical vibration effects [40]. Under ultrasonic stimulation, these hydrogen-producing metallic nanomaterials utilize ultrasound (frequency range: 0.5–3 MHz) to activate photosensitizers concentrated in tumor tissue, causing them to generate H_2_ and ROS. The therapeutic molecules work in synergy with mechanical vibrations and cavitation effects to kill cancer cells while minimizing damage to surrounding healthy tissue. Currently, the design and optimization of acoustic-driven hydrogen-producing metallic nanomaterials often follow approaches similar to those used for piezoelectric materials. By designing metallic spatial structures (e.g., non-centrosymmetric structures) or crystal phases [41], introducing semiconductor materials (e.g., TiO_2_), constructing heterojunctions [42], and adding metals such as Bi and Ga [43], the efficiency and stability of the acoustic-sensitive agents can be controlled. This enables electronic transitions under ultrasonic stimulation, driving reactions with H_2_O or H_2_O_2_ to produce H_2_ and ROS. Among these, liquid metals (LM) have gradually emerged as a frontier in piezoelectric catalysis research due to their unique dynamic atomic rearrangement, high electrical conductivity, and excellent deformability, which confer significant delivery and catalytic capabilities. A catalytic system, Ga_67_In_20.5_Sn_12.5_@tumor cytomembranes (LM@M) [44], utilizes piezoelectric catalysis to decompose water in the tumor stroma to generate H_2_, thereby activating the cGAS-STING immune pathway, enhancing antigen presentation, and promoting T-cell infiltration into deep tumor regions. Its dynamic deformation capability significantly improves the system’s penetration efficiency and enables the elimination of primary and distant metastatic tumors in mice. Notably, the introduction of ultrasound generates microbubbles in liquid environments, triggering a cavitation effect that synergizes with ROS to induce cellular calcium influx and calcium overload. This activates the endoplasmic reticulum PERK stress signaling pathway, releasing immunogenic signals that promote dendritic cell maturation and antigen presentation, thereby initiating an adaptive immune response [45]. Concurrently, H_2_ selectively removes excess ATP and ROS from the tumor microenvironment, preventing oxidative stress-induced damage to dendritic cells (DCs) and effector T cells, thereby achieving regulation of the tumor microenvironment.

### 2.4. Electrically Responsive Metallic Nano-Materials

In 2019, Gu et al. [46] successfully eliminated tumors larger than 500 mm^3^ by using a square-wave alternating electric field to drive platinum nanoparticles (PtNPs) to hydrolyze and generate hydroxyl radicals (·OH). Since then, electrodynamic therapy (EDT) has garnered significant attention in cancer treatment. Compared to electrochemotherapy (EChT), which involves the invasive insertion of electrodes into tumors to kill cancer cells under direct current, EDT uses platinum nanoparticles as its core material, making it a more minimally invasive and controllable approach. Furthermore, unlike other stimulus-responsive materials, EDT does not rely on oxygen; it directly utilizes electric fields to hydrolyze water molecules, generating ·OH, H_2_ and O_2_ [47], thereby overcoming the hypoxic tumor microenvironment. Researchers often nano-engineer, porosify, and composite Pt to achieve high material penetration, high electron transfer efficiency, and excellent stability [48]. The ·OH, H_2_ and O_2_ generated by this strategy can both induce ICD and reshape the redox homeostasis of the tumor microenvironment, effectively addressing hypoxia-induced suppression in the tumor microenvironment [49]. This approach can be combined with chemotherapy and immunotherapy to promote highly effective tumor treatment.

## 3. Mechanisms of Antitumor Immune Regulation in Nanometal Hydrogen Therapy and Three-Dimensional Structure–Activity Relationships

The immune regulation achieved through nanometal hydrogen therapy dynamically modulates the tumor microenvironment via the actions of H_2_, O_2_, ROS, and metal ions. It addresses the various immune evasion mechanisms employed by tumors through three key mechanisms: enhancing immune balance [50], reshaping immune surveillance [16], and synergizing immune responses [51], thereby combating tumors at their root. In this process, the structure–activity relationship between “metal type-specific activity-immune effect” often influences H_2_ production rates, mechanisms of action, molecular pathways, immune regulatory effects and antitumor efficacy. Metal nanomaterials exhibit distinct physicochemical properties (such as stability, response mechanisms, and catalytic activity) based on their elemental composition and crystal structure. These properties determine the specific activities of the materials in generating H_2_ or oxygen in solid tumors, regulating ROS, and adjusting the pH of the tumor microenvironment [52,53]. By leveraging these specific activities to activate or inhibit molecular pathways, they suppress cancer cell immune evasion and achieve targeted killing of cancer cells through immune effects such as ICD activation, M1/M2 polarization, and immune cell infiltration [54,55]. In the metabolic fate of cancer cells, the accumulation of H_2_ disrupts the redox balance within cancer cells, while the immune effects activated by hydrogen therapy can address intratumoral heterogeneity through immune editing, thereby systematically blocking the dynamic process of tumor immune evasion and enhancing antitumor efficacy.

### 3.1. Hydrogen Immunotherapy: Enhancing Immune Balance and Reversing the Immunosuppressive Microenvironment

The root cause of the difficulty in completely curing solid tumors lies in their ability to evade immune surveillance and resist immunotherapy by establishing an immunosuppressive microenvironment, which reduces treatment efficacy [56]. This microenvironment consists of the dense extracellular matrix (ECM), the physical barrier caused by high interstitial fluid pressure, and the biochemical barrier characterized by hypoxia and acidification, all of which severely inhibit the infiltration and activity of cytotoxic T cells and NK cells [57,58]. Therefore, reversing the immunosuppressive microenvironment and reactivating the cancer immune cycle represent the primary breakthroughs in anticancer therapy [59]. Nanometal hydrogen therapy utilizes stimulus-responsive nanomaterials to generate hydrogen in situ, thereby inhibiting tumor glycolysis. Simultaneously, the OH^−^ and O_2_ work synergistically to improve the hypoxic and acidified microenvironment, thereby lifting immunosuppression and restoring immune cell function.

In the process of reversing the immunosuppressive microenvironment, nanometal materials often generate H_2_ in situ in response to electrical, optical, ultrasonic and acidic microenvironmental stimuli, thereby protecting immune cells from damage caused by high ATP levels and strong oxidative conditions in the microenvironment [38]. Simultaneously, influenced by the core metal, these materials produce active factors such as OH^−^, O_2_, ROS, and metal ions, which activate complex apoptosis or pyroptosis signaling pathways, thereby enhancing the infiltration capacity of immune cells and their antitumor efficacy [60].

**Figure 2:**
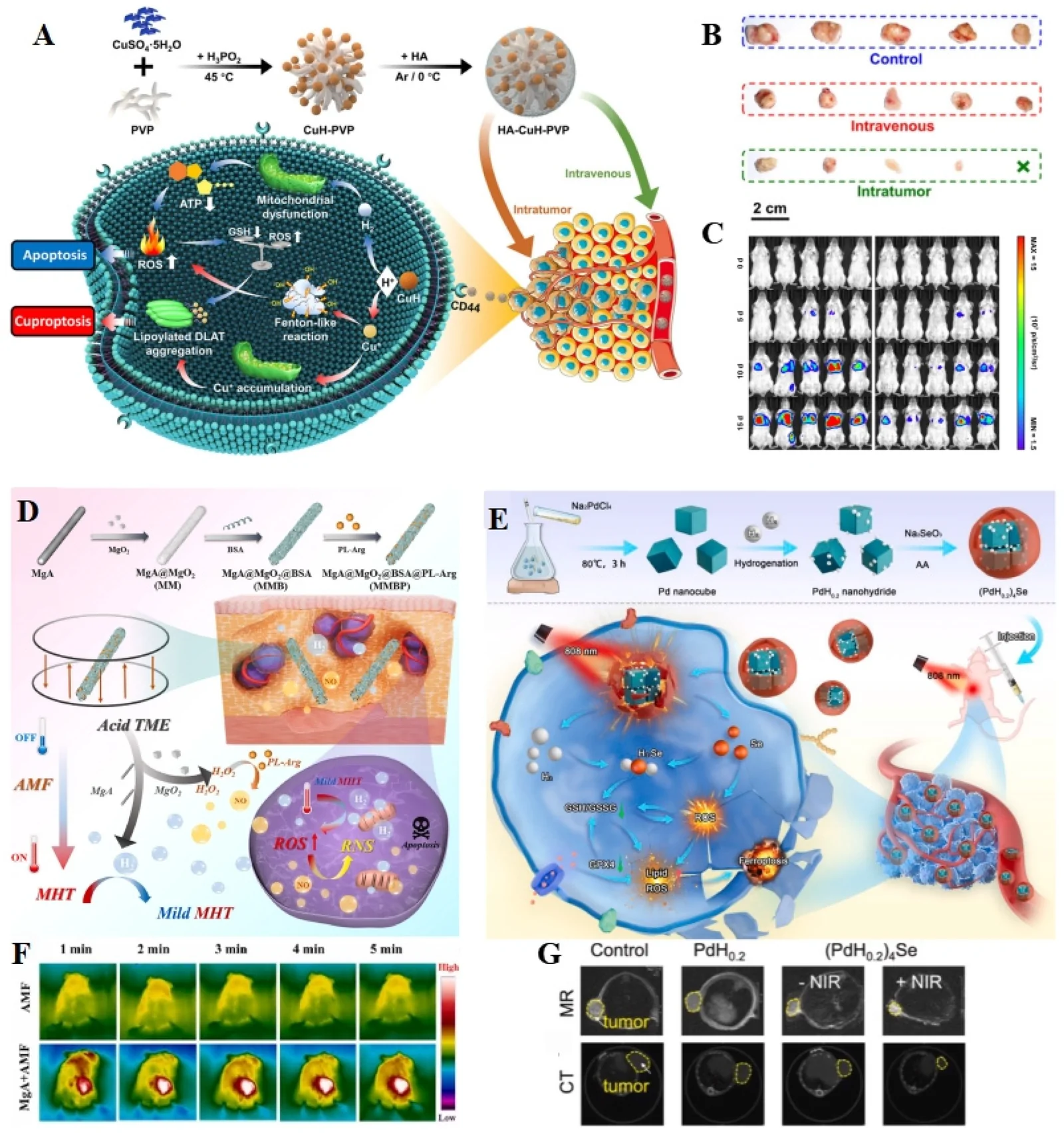
Hydrogen-producing nanometal materials that reverse the immunosuppressive microenvironment. (**A**) Synthesis pathway and therapeutic mechanism of the HCP nanoplatform for tumor treatment [25]. (**B**,**C**) Comparison of the therapeutic effects of HCP on 4T1 tumors and 4T1 lung metastases [25]. (**D**) Schematic of the MMBP material synthesis process and the mechanism of H_2_/MMHT for tumor treatment [62]. (**F**) Thermal imaging showing the rapid increase in intratumoral temperature in mice under an alternating magnetic field [62]. (**E**) Synthesis route of the (PdH_0.2_)_4_Se nanotetramer and the ferrocide-mediated anticancer mechanism of (PdH_0.2_)_4_Se following NIR irradiation [34]. (**G**) Tumor localization map after (PdH_0.2_)_4_Se treatment in mice [34].

The hydrogen-mediated immune pathway, centered on enhancing immune balance, reveals a unique structure–activity relationship between “metal type-specific activity-immune effects” (Table 1). From the materials design perspective, it is evident that such metal nanomaterials primarily utilize Mg, Cu, Pt, Pd and Bi as their core metals. In particular, Mg and Cu often respond to acidic microenvironments by releasing H_2_ through hydrolysis to disrupt mitochondrial function in cancer cells. Mg(OH)_2_ neutralizes the acidic TME to reduce immunosuppression, while Cu^2+^ promotes GSH depletion and induces copper-mediated cell death [61], thereby reshaping the tumor microenvironment. Guan et al. developed hyaluronic acid-coated CuH nanoparticles (HA-CuH-PVP) using microfluidic technology. Upon cellular uptake, the material rapidly degrades under acidic conditions, releasing Cu^2+^ and generating H_2_ in situ (Figure 2A). Due to a Fenton-like reaction involving peroxidase-like (POD-like) Cu^2+^ and ATP downregulation caused by excess H_2_, the ability to induce cancer cell apoptosis is synergistically enhanced (Figure 2B,C) [25]. Li et al. engineered a magnesium alloy rod (MgA@MgO_2_@BSA@PL-Arg, MMBP) by combining H_2_ and magneto-hyperthermia (MMHT) technology, which was implanted into residual tumor tissue sites post-surgery (Figure 2D). MMBP undergoes rapid degradation in an acidic tumor microenvironment (approximately 70% mass loss within 90 days) but degrades slowly in normal tissue (less than 20% mass loss after 90 days), demonstrating excellent biocompatibility [62]. At the same time, the magnesium alloy continuously generates H_2_ in an acidic environment, which not only protects surrounding normal tissues but also assists the magnesium alloy in achieving MMHT under the action of a low-intensity alternating magnetic field (AMF) (tumor tissue temperature exceeding 45 °C for 5 min after implantation) (Figure 2F). The unique feature of this material lies in the fact that the large amount of ROS and NO (generated by the action of BSA and PL-Arg) produced under high-temperature stimulation combine to form an active nitrogen species (RNS) storm, which can jointly inhibit tumor recurrence with H_2_. Most studies still focus on the fact that H_2_ reverses the tumor microenvironment by neutralizing the acidic environment. However, some research has found that H_2_ can alleviate oxidative stress in cancer-associated fibroblasts (CAFs) [63], thereby reshaping the phenotype of CAFs and inhibiting the release of pro-tumor and immunosuppressive factors by CAFs. This study (Mg-CaCO_3_) innovatively addresses tumor immunosuppression by targeting the physical tumor barrier, providing a new theoretical basis for the diversified mechanisms by which H_2_ reshapes tumor immune editing [23].

In contrast, Pt and Pd enable on-demand hydrogen production under external stimulation via electrocatalysis or photocatalysis. However, the poor biodegradability of precious metals and the high risk of accumulation in the body make their biosafety a concern that cannot be ignored. Building on traditional Chinese acupuncture techniques, Jin’s team developed a locally tumor-selective electrochemical therapy (H_2_-ETC) in which iron needles were implanted into mouse tumors as anodes [64]. A 3 V voltage was applied to electrolyze water and produce H_2_ within the tumor’s acidic microenvironment. This study quantitatively controlled H_2_ production by applying a voltage (at pH 7.4 and 6.0, hydrogen production increased rapidly with rising voltage), providing a viable on-demand therapeutic strategy to circumvent the issues of gas diffusion and uncertain accumulation levels. Meanwhile, Pd and its hydrides [65], owing to strong H_2_ storage capacity and autocatalytic hydrogenation ability, activate plasmonic resonance effects under NIR illumination, thereby generating heat and H_2_. The high temperature and H_2_ induce redox stress in cancer cells, inhibiting their glucose degradation [66]. A high-hydrogen-storage nanotetramer (PdH_0.2_)_4_Se (about 45 nm) based on a palladium-selenium core accumulates within tumors via enhanced permeation and retention (EPR) and achieves controlled spatiotemporal H_2_ release upon stimulation by NIR light (808 nm) [34]. Simultaneously, SeH_2_ generated by the reaction of H_2_ with Se effectively disrupts the GSH/GSSG ratio (Figure 2E,G) and significantly downregulates GPX4 expression in SLC7A11 cells, thereby inducing ferrocytosis and thereby significantly inhibiting cancer cell proliferation, migration, invasion, and angiogenesis. Furthermore, a comparison of Pd, PdH_0.2_, and (PdH_0.2_)_4_Se at the cellular level revealed that Pd caused a significant increase in ROS levels in both normal liver cells and liver cancer cells, whereas PdH_0.2_ and (PdH_0.2_)_4_Se had no effect on ROS levels in normal cells but significantly increased ROS levels in hepatocarcinoma cells. This suggests that the presence of H_2_ can alter the selectivity of ROS induction by nanomaterials, amplifying oxidative stress in tumor cells. However, the net contribution of H_2_ remains difficult to determine.

It is worth noting that most nanoplatforms, while producing hydrogen, are also associated with the release of metal ions (such as the Cu^2+^-induced Fenton reaction and Mn^2+^-activated cGAS-STING), thermal effects (photothermal or magnetothermal), and the generation of reactive molecules (O_2_, ·OH, RNS). Due to these multiple factors, the immune-activating effects cannot be attributed solely to H_2_. At the same time, the vast majority of studies on hydrogen-producing nanometals in the tumor microenvironment struggle to establish “non-hydrogen-producing material” controls; consequently, it is difficult to quantitatively assess the role of H_2_ in therapy, and it remains unclear whether H_2_ acts directly on a specific protein target within signaling pathways or indirectly alters the activation state of these pathways by scavenging ROS. However, the aforementioned studies demonstrate that H_2_ production may maintain immune cell viability and protect normal cells by scavenging excess ROS and regulating mitochondrial function through its antioxidant properties. Furthermore, although some studies have linked H_2_ to cGAS-STING activation, there is currently no direct evidence proving that H_2_ can physically interact with cGAS, STING, or any other protein. H_2_ may indirectly create favorable conditions for the cGAS-STING pathway by regulating mitochondrial function.

In this hydrogen-based immune regulatory system, metal nanomaterials catalyze water splitting to produce H_2_. H_2_ disrupts mitochondrial function in cancer cells and protects immune cells, while the O_2_ generated by the reaction, along with metal ions and their hydroxides, regulates the tumor microenvironment. Synergistically, these two mechanisms reverse the immunosuppressive microenvironment and increase CD8^+^ T cell infiltration. Therefore, when discussing hydrogen-mediated immunology, most current articles rigidly distinguish between the specific immunological effects of H_2_ and the nonspecific effects of nanomaterials, overlooking the possibility that cascading effects during the hydrogen-producing process of nanomaterials (such as metal ions, thermal responses, and reactive molecules) may jointly or sequentially activate signaling pathways. In particular, the hydrogen-production process in TEM-responsive materials is uncontrollable, which has led to relatively limited research on their mechanisms and makes it difficult to demonstrate the activating or enhancing role of H_2_ in immune responses through experimental comparisons under controlled activation conditions. Furthermore, there is currently a lack of comparative experiments to demonstrate that a particular metal is indeed superior to other alternatives under the same conditions.

**Table 1:** Summary of Hydrogen-Producing Metal Nanomaterials for Cancer Therapy via Reversal of the Immunosuppressive Microenvironment.

Metal Nanomaterials	Cancer Model	Size	Biodegradability and Toxicity	Response Conditions	Active Molecules	Mechanism of Action	Efficacy	Route of Administration	Dosage	Clinical Readiness
MgG rod (Mg-Pt alloy) [50]	4T1 and CT26 tumor models; VX2 rabbit liver orthotopic tumor mode	3 nm Pt	High; biodegradable; no bioaccumulation; no significant toxicity.	Bioelectricity	H_2_, Mg(OH)_2_	Mg(OH)_2_ neutralizes the acidic tumor microenvironment. H_2_ induces mitochondrial dysfunction, disrupting the redox homeostasis of cancer cells.	MgG significantly prolonged survival in mice and rabbits.	Intratumoral implantation	Mice: 2 MgG rods (D = 0.5 mm, L = 4 mm) Rabbits: 3 MgG rods (L = 8.0 mm, D = 0.8 mm) (about 12 mm × 12 mm)	Animal Testing Phase
Pt-Bi_2_S_3_ [60]	4T1 tumor model	Diameter: 20 nm; length: 100 nm	Moderate; accumulates in the liver, tumors, and kidneys; toxicity has not been established.	Ultrasound stimulation (1 MHz, 50% duty cycle, 1.5 W cm^−2^, 10 min)	H_2_, O_2_, ROS	Pt-induced oxygen production alleviates hypoxic conditions, while H_2_ production promotes GSH depletion, thereby reversing the tumor-suppressive immune microenvironment.	The treatment group effectively inhibited tumor growth.	Intravenous injections	100 μL 10 mg kg^−1^	Animal Testing Phase
Bi_2_Te_3_-Au [67]	Hepa1–6 tumor model	500 nm BT; 15 nm Au	High; no bioaccumulation; no significant toxicity.	Ultrasound stimulation (1 MHz, 0.2 W cm^−2^, 20 min, 4 × 5 min)	H_2_, H_2_O_2_	\	The tumor eradication rate in the treatment group was 100% (28 days).	Intravenous injections	10 mg kg^−1^	Animal Testing Phase
CuH [25]	4T1 tumor model	60 nm	High; acid-degradable; no significant toxicity.	TME (acidic pH)	H_2_, H_2_O_2_, Cu^2+^	Synergistic cytotoxic effects resulting from H_2_-induced GSH depletion and Cu^2+^-mediated Fenton reactions.	Tumor suppression rate in the treatment group was ≥90% (15 days).	Intravenous injections	20 mg kg^−1^	Animal Testing Phase
Cu@CDCN [68]	4T1 tumor model	100 nm	High; no significant toxicity.	Sunlight (400–500 nm, 30 mW cm^−2^, 15 min)	H_2_, H_2_O_2_, Cu^2+^	H_2_ and Cu^2+^ jointly induce mitochondrial dysfunction, GSH depletion, and disruption of the redox homeostasis in cancer cells.	The treatment group significantly suppressed tumor growth (14 days).	Intravenous injections	10 mg kg^−1^	Animal Testing Phase
(PdH_0.2_)_4_Se [34]	SGC-7901 tumor model	20 nm	High; biodegradable; no bioaccumulation; no significant toxicity.	Light sensitivity (808 nm, 1 W cm^−2^)	H_2_, H_2_Se	H_2_ depletes GSH and promotes ROS production, while (PdH_0.2_)_4_Se induces lipid peroxidation, synergistically inducing ferroptosis in cancer cells.	\	Intravenous injections	100 μL 2 mg mL^−1^	Animal Testing Phase
Mg-CaCO_3_ [23]	MC38 and 4T1 tumor models	40–80 nm CaCO_3_; 4 mm Mg-CaCO_3_	High; biodegradable; no bioaccumulation; no significant toxicity.	TME (acidic pH)	H_2_, ROS	H_2_ induces the accumulation of ROS and depletion of GSH within tumors, inhibits CAFs, and remodels the TME.	Primary tumor suppression rate: 4T1: 54.34%, MC38: 77.15%.	Intratumoral implantation	1 per piece	Animal Testing Phase
M-TACE (Lip Mg-TACE) [69]	H22 tumor model; VX2 rabbit liver orthotopic tumor model	20 μm	High; biodegradable; no bioaccumulation; no significant toxicity.	TME (acidic pH)	H_2_, Mg(OH)_2_	Mg(OH)_2_ neutralizes the acidic tumor microenvironment, while H_2_ reverses immune suppression and triggers a specific T-cell-mediated antitumor response; together, these synergistic effects inhibit tumor growth.	The objective tumor response rate reached as high as 93.3%.	Transarterial chemoembolization (TAE)	Mice: 25 μL 80 mg mL^−1^ Rabbits: 0.3 mL 80 mg mL^−1^	Animal Testing Phase
MgA@MgO_2_@BSA@PL-Arg(MMBP) [62]	Luc-4T1 tumor model	600 nm	High; biodegradable; no bioaccumulation; no significant toxicity.	TME (acidic pH)	H_2_, RNS	H_2_ protects normal tissue, while the reactive nitrogen species (RNS) generated by mild magnetic hyperthermia (MMHT) kill cancer cells.	\	Surgical implantation	\	Animal Testing Phase
MgNF@PEG/PMNVP [70]	MCF-7 and MC38 tumor models	65 nm	High; biodegradable; no bioaccumulation; no significant toxicity.	TME (acidic pH)	H_2_	H_2_ not only damages mitochondria, leading to a decrease in cellular ATP levels, but also induces high oxidative stress and disrupts the intracellular redox balance, severely damaging cellular DNA and resulting in cell apoptosis.	\	Intravenous injections	100 μL 2 mg mL^−1^	Animal Testing Phase
PdH NCs [71]	4T1 tumor model	20–30 nm	High; no bioaccumulation; no significant toxicity.	TME (acidic pH)	H_2_, GOx, ·OH, O_2_	GOx depletes glucose to produce ROS, which induce DNA damage and lipid peroxidation via ·OH radicals. Meanwhile, H_2_ enhances CAT-like activity, alleviates hypoxia, and increases sensitivity to oxidative stress.	\	Intravenous injections	10 mg kg^−1^	Animal Testing Phase
CoB@PDA [72]	HepG2 tumor model	494.92 nm	High; no bioaccumulation; no significant toxicity.	Light sensitivity (808 nm, 0.56 W cm^−2^, 10 min); TME (acidic pH)	H_2_, O_2_, ·O^2−^, ^1^O_2_	H_2_ has potent anti-inflammatory effects, while Co^2+^ and ROS promote DNA denaturation and damage, inducing apoptosis.	The tumor was completely eliminated within 14 days.	Intratumoral injection	2 mg mL^−1^	Animal Testing Phase
ZrTc-Co@HA(ZTCH) [33]	H22 tumor model	210 ± 10 nm	Moderate; toxicity has not been established; The plasma half-life of this drug is 2.26 h.	Light sensitivity (1250 nm, 0.1 W cm^−2^, 5 min)	H_2_, ROS	H_2_ targets mitochondria, impairing mitochondrial function by inhibiting adenosine triphosphate (ATP) synthesis, thereby inducing apoptosis.	The tumor suppression rate in the treatment group reached 95%.	Intravenous injections	5 mg kg^−1^	Animal Testing Phase
H_2_-ECT [64]	C6 tumor model	\	High; biodegradable; no significant toxicity.	Electrical stimulation (3 V, 10 min); TME (acidic pH)	H_2_	H_2_ impairs mitochondrial function and induces apoptosis.	\	Intratumoral implantation	1 per piece	Animal Testing Phase
Carbon/potassium-doped red polymeric carbon nitride (RPCN) [73]	4T1 tumor model	\	Moderate; No visible damage to any organs.	Light sensitivity (808 nm, 0.5 W cm^−2^, 20 min)	H_2_, ROS	H_2_ impairs mitochondrial function, while ROS depletes GSH, and together they induce apoptosis.	The tumor completely disappeared 21 days after treatment.	Intratumoral injection	60 µL 2 mg mL^−1^	Animal Testing Phase
PCN-224@Pd/H_2_ [74]	MDA-MB-231 tumor model	\	Moderate; no significant toxicity; slight hemolysis.	Light sensitivity (660 nm, 100 mW cm^−2^, 10 min)	H_2_, ^1^O_2_	H_2_ induces redox stress, causing damage to cancer cells.	The tumor suppression rate in the treatment group reached 84.8%.	Intravenous injections	100 µL 15 mg kg^−1^	Animal Testing Phase
DFA IV-PEI-PEG-GNRs [49]	CT26 tumor model	60.60 ± 2.7 nm	High; biodegradable; no significant toxicity.	Electrical stimulation (1.0 mA, square-wave DC potential of 4.3 V cm^−1^)	H_2_, O_2_, ROS	H_2_ and O_2_ work synergistically to alleviate hypoxia and acidity in the tumor microenvironment. Intracellular calcium accumulation mediated by endoplasmic reticulum stress, coupled with ROS production, induces cancer cell apoptosis.	The survival period of the treated group of mice was extended to 45 days.	Intratumoral implantation	50 μg mL^−1^	Animal Testing Phase
multifunctional Cu-doped ZnS nanocatalyst (ZnS:Cu) [35]	4T1 tumor model	60–80 nm	High; no significant toxicity.	Light sensitivity (1060 nm, 0.75 W cm^−2^, 5 min)	H_2_	H_2_ induces redox stress, promotes GSH depletion, activates tumor immunity, and triggers ferroptosis in cancer cells.	The treatment group achieved complete tumor elimination, with mice surviving up to 60 days.	Intravenous injections	100 µL 10 mg kg^−1^	Animal Testing Phase
mPDAB [75]	4T1-luc tumor model	100 nm	High; no significant toxicity.	TME (acidic pH); Light sensitivity (808 nm, 1.5 W cm^−2^, 6 min)	H_2_, ROS	High-temperature NIR induces apoptosis and necrosis in cancer cells, while ROS disrupts the redox homeostasis of tumor cells, thereby killing the tumors. At the same time, H_2_ scavenges excess ROS to alleviate the inflammatory response.	\	Intravenous injections	30 mg mL^−1^	Animal Testing Phase

### 3.2. Hydrogen-Mediated Immunity: Reshaping Immune Surveillance and Inducing Immunogenic Cell Death

Current clinical treatment strategies (including surgery, radiation therapy, chemotherapy, targeted therapy, and immunotherapy) have limited efficacy against complex tumors [76] due to issues such as abnormal tumor vascularization, tumor heterogeneity, adverse effects, and drug resistance. Although immune surveillance serves as the body’s primary line of defense against tumors [77], immunosuppressive cells (such as Tregs, CAFs, and TAMs) present in the tumor microenvironment compete with tumor cells for nutrients and induce a state of immune silencing, making it difficult for the body’s immune system to eliminate the tumor [78]. The key to restoring immune surveillance lies in disrupting the tumor-immune interaction network and reprogramming cancer cells [79]. Nano-metal hydrogen therapy uses H_2_ to induce mitochondrial dysfunction and oxidative stress in tumor cells, promoting immunogenic cell death (ICD) and releasing damage-associated molecular patterns (DAMPs) and tumor-associated antigens (TAAs), which in turn promotes the maturation of dendritic cells (DCs), breaks immune tolerance, and reactivates the antitumor immune response [80].

**Figure 3:**
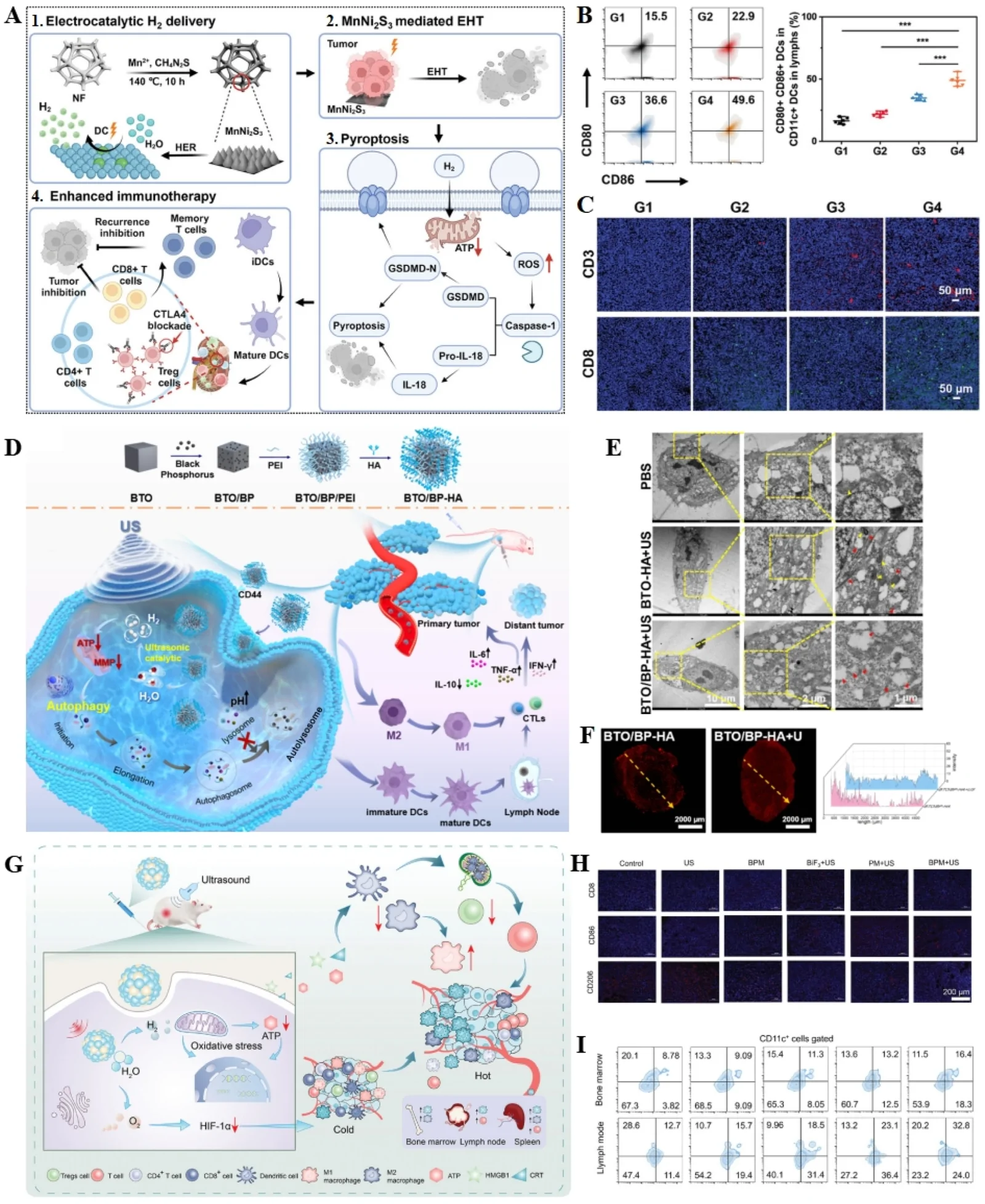
Hydrogen-producing nanomaterials capable of modulating immune surveillance. (**A**) Preparation of MnNi_2_S_3_ nanoelectrodes and the mechanism by which they mediate electrocatalytic hydrogen-induced immune enhancement and T-cell infiltration [86]; (**B**,**C**) Quantitative analysis of mature DCs and immunofluorescent staining for CD3 and CD8 in tumor tissue sections treated with MnNi_2_S_3_ and other methods [86]. *** *p* < 0.001. and the data are presented as the mean ± SD; (**D**) Schematic illustration of sonocatalytic BTO/BP-HA-mediated tumor cell autophagy to enhance immune responses [95]; (**E**) TME observations of cellular autophagy in the BTO-HA + US and BTO/BP-HA + US groups (yellow: lysosomes; red: autophagosomes) [95]; (**F**) Fluorescence images and quantitative analysis of BTO/BP-HA distribution in tumors (red: RB) [95]; (**G**) Schematic diagram of the mechanism by which Mo_2_C-POM-BiF_3_ (BPM) activates immune responses via sonocatalytic H_2_ production [43]; (**H**) Immunofluorescence staining of tumor tissue sections for CD8, CD86, and CD206 treated with BPM and other methods [43]; (**I**) Quantitative analysis of mature DCs in bone and lymphoid tissues treated with BPM and others [43].

Restoring immune surveillance is a multidimensional activation process. On the one hand, it involves suppressing the function of immunosuppressive cell populations, such as Tregs, and restoring an environment conducive to immune cell infiltration. On the other hand, it requires inducing ICDs to restart the antigen-presentation cycle [81]. Nano-metal materials respond to external stimuli based on their chemical properties such as electronic structure and redox potential. By mimicking biological enzymes, releasing metal ions, generating ROS, depleting GSH, and producing H_2_/O_2_, they overcome immune suppression barriers. This includes reversing the function of immunosuppressive cells, inducing ICD, limiting tumor glycolysis, and restoring the metabolic function of normal immune cells [82]. At the same time, they mobilize specific molecular pathways through signaling molecules, such as cGAS-STING, PD-1/PD-L, NF-κB and PI3K/Akt, to block immune-suppressing signals at the signaling level and jointly achieve the tumor immune activation state [83]. In Mn-based materials, the multivalent nature of Mn (Mn^2+^/Mn^3+^/Mn^4+^) confers redox activity and enzyme-like catalytic capabilities (including superoxide dismutase and catalase). Notably, the Mn^2+^ generated by GSH depletion in these materials can potently activate the cGAS-STING pathway to secrete type I interferons and induce adaptive immunity [84]. Furthermore, the enzymatic catalytic activity and hydrogen-producing properties of manganese dynamically regulate the generation and clearance of ROS, activating the p38/MAPK axis within macrophages and inducing the polarization of M2-type TAMs toward the M1 phenotype [85]. This enhances antigen presentation capacity during immune surveillance and improves the efficiency of subsequent immune cell activation and infiltration. Nickel sulfides such as Ni_2_S_3_ are stable under acidic conditions and facilitate H_2_ adsorption and desorption during electrolysis. Li et al. demonstrated that doping Ni_2_S_3_ with Mn can modulate the electronic structure of Ni atoms to enhance the hydrogen evolution reaction (HER) activity of the electrode. Simultaneously, Mn^2+^ reverses the immunosuppressive microenvironment (Figure 3A) [86]. Under low-voltage catalysis, the construction of MnNi_2_S_3_ NEs can sustainably produce large amounts of H_2_, induce mitochondrial dysfunction, activate pyroptosis via the ROS/caspase-1/GSDMD signaling pathway, release damage-associated molecular patterns (DAMPs) to promote DC maturation, and further activate effector T cells. Furthermore, MnNi_2_S_3_ nanoparticle-mediated electric field thermotherapy (ETH) synergistically enhances the infiltration of CD8 T cells into tumor tissue. The high-level immune response induced by this material achieves a 100% suppression rate of CT26 tumors (Figure 3B,C).

Mg- and Ca-based materials leverage the properties of spontaneous H_2_ production and the alkalinity of hydroxides to first improve the acidic tumor microenvironment, and then synergistically activate immune surveillance through H_2_ and Mg^2+^/Ca^2+^ via different pathways: (1) The therapeutic effects of H_2_ exhibit dose-dependence and tissue specificity [87]. In tumors, high concentrations of H_2_ can directly inhibit mitochondrial respiration, hinder ATP synthesis, and disrupt cancer cell energy metabolism. Simultaneously, it induces ROS accumulation and disrupts the redox homeostasis of the inner mitochondrial membrane (IMM) [88], thereby mediating pyroptosis via the ROS/NLRP3 inflammasome/caspase-1/GSDMD pathway to mediate pyroptosis and via the activation of the caspase-9/caspase-3 or ROS/NF-κB/p53 signaling axis to induce apoptosis, releasing tumor antigens and inflammatory factors (such as IL-1β and IL-18), which are then recognized and presented by DCs and other cells, thereby initiating an adaptive immune response. In normal cells or immune cells, H_2_ at certain concentrations exerts antioxidant and protective effects by scavenging excess ROS, thereby protecting normal cells and immune cells from oxidative damage [89]. (2) Mg^2+^ and Ca^2+^ can serve as immune signaling molecules that are actively sensed and responded to by immune cells. Specifically, Mg^2+^ enhances T-cell activation and amplifies immune effects by regulating the NF-κB signaling pathway, binding to lymphocyte function-associated antigen-1 (LFA-1) on the T-cell surface [90], activating the T-cell receptor (TCR)-ITK pathway via influx [91], and inducing T-cell differentiation through the TRPM7-Mg^2+^ axis [92]. Generally, higher intracellular Mg^2+^ levels are more conducive to activating the immune system and enhancing the cytotoxic capacity of immune cells. In contrast, Ca^2+^ regulates the activation and differentiation of immune cells (such as T cells, B cells and DCs) by modulating the SOCE-NFAT pathway through calcium channel activation. Gong et al. prepared nano-CaH_2_ using liquid-phase exfoliation, dispersed it in Lipiodol, and developed a nano-CaH_2_-lipiodol composite embolization agent for the treatment of in situ liver cancer in rabbits via transarterial embolization (TAE) [93]. Through a “combined approach” involving “Ca^2+^-induced calcium overload, H_2_-mediated inhibition of mitochondrial function, OH^−^-mediated neutralization of the acidic microenvironment”, it induced ICD, activated DC maturation, and promoted CD4^+^ and CD8^+^ T cell infiltration, thereby significantly improving the therapeutic effect of iodized oil embolization.

At the same time, sonodynamic therapy (SDT) benefits from low-intensity ultrasound’s deep penetration, minimal energy attenuation, and the acoustic cavitation effect, which significantly enhance its therapeutic safety, versatility, and patient tolerance. Ba and Bi are the primary metals in ultrasonic catalytic systems. Under ultrasonic stimulation, these materials generate a piezoelectric electric field that drives the decomposition of water within tumors, producing ROS, H_2_ and O_2_ [94]. O_2_ alleviates the hypoxic tumor microenvironment and reverses the immunosuppressive environment, while H_2_ disrupts mitochondrial function in cancer cells, inducing ICD and thereby activating DCs to perform antigen recognition and presentation. To enhance therapeutic efficacy, researchers often combine BaTiO_3_ (BTO) with compounds such as Mg and black phosphorus (BP) to improve the H_2_ and ROS production capabilities of these materials. When BTO/BP-HA is applied to cells via sonication, it activates autophagy in U14 cells, leading to a significant intracellular accumulation of the protein p62, which in turn inhibits lysosomal degradation (Figure 3E) [95]. Concurrently, this material catalyzes the hydrolysis of water in the tumor interstitial fluid to generate H_2_, thereby reducing tumor interstitial pressure (TIP) to enhance deep delivery of nanoparticles (Figure 3F). It also promotes tumor cell apoptosis by inhibiting mitochondrial respiration, induces ICD, and activates DC maturation and effector T-cell infiltration (Figure 3D). Notably, the addition of BP achieves a dual regulatory function: on the one hand, it forms a heterojunction to accelerate electron–hole separation and thus rapidly produce H_2_; on the other hand, it inhibits cellular autophagy mechanisms to reduce the cytotoxicity of the nanoparticles, while simultaneously degrading to form PO_4_^3−^ within lysosomes to increase lysosomal pH, further enhancing the immune response of this material [96]. Meanwhile, the ternary Schottky junction photocatalyst BPM (Mo_2_C-POM-BiF_3_), composed of BiF_3_, polyoxometalate (POM), and Mo_2_C, utilizes POM in various valence states as an electron transfer medium. BiF_3_ and Mo_2_C form a Schottky barrier and enhance the separation and migration efficiency of charge carriers, thereby catalyzing the decomposition of water into H_2_ and O_2_ under ultrasonic stimulation [43]. Specifically, O_2_ alleviates the hypoxic tumor microenvironment, H_2_ disrupts mitochondrial function and induces DNA damage, while BiF_3_ depletes GSH to disrupt the tumor’s redox homeostasis, thereby promoting cancer cell apoptosis through multiple mechanisms and inducing ICD (Figure 3G). Furthermore, DAMPs released by ICD, such as calreticulin (CRT) and high-mobility group box 1 (HMGB1), promote the maturation of DCs, activate CD4^+^/CD8^+^ T lymphocyte infiltration, and repolarize TAMs from the immunosuppressive M2 phenotype to the pro-inflammatory M1 phenotype (Figure 3H,I), thereby reshaping immune surveillance against the tumor.

Hydrogen-producing nanomaterials disrupt tumor immune silencing by modulating the acidity, hypoxia, and immunosuppressive populations within the TME. They then synergistically promote the activation of immune cells through hydrogen-mediated or multi-mechanistic effects to enhance the immune response and restore immune surveillance against tumors, thereby effectively combating tumors through both apoptosis induction and long-term immune effects (Table 2).

### 3.3. Hydrogen Immunotherapy: Activates the Systemic Immune System and Enhances the Efficacy of Chemotherapy and Radiation Therapy

Although radiation therapy and chemotherapy are the primary treatments for cancer and offer excellent efficacy, their long-term use can easily lead to primary or secondary drug resistance and significant toxic side effects, which severely impact patients’ quality of life and median survival [99]. H_2_, with its unique ability to regulate cells, can enhance sensitivity to radiation and chemotherapy while reducing damage to normal cells [100]. On the one hand, H_2_ induces oxidative stress in tumor cells by binding to iron porphyrins to cause mitochondrial dysfunction and generate ROS, or by depleting GSH and disrupting the GSH-Nrf2 axis; simultaneously, as a signaling molecule, it inhibits the PI3K/Akt and NF-κB pathways while activating the MAPK pathway, synergistically inducing tumor cell cycle arrest and apoptosis [101]. On the other hand, H_2_ selectively scavenges ·OH and ONOO^−^, reduces the levels of inflammatory factors such as IL-6, regulates apoptotic proteins such as caspase-3, and alleviates inflammation and damage to normal cells caused by radiotherapy and chemotherapy; at the same time, by improving mitochondrial function, it promotes the activation of CD4^+^ and CD8^+^ T cells, protects immune function, and prolongs the duration of antitumor efficacy [102].

**Figure 4:**
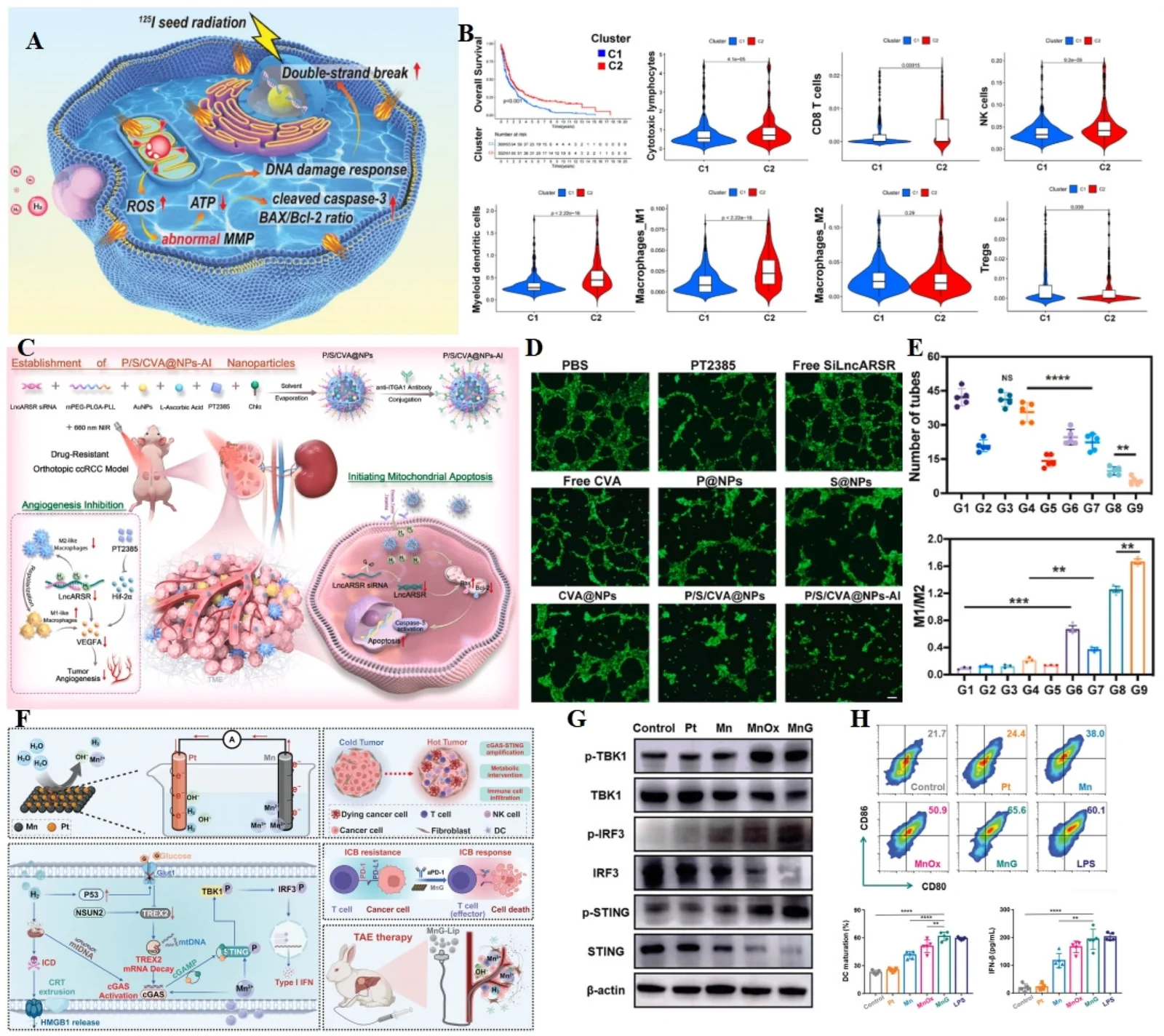
Hydrogen-producing nanomaterials that enhance the efficacy of anticancer drugs and activate systemic immunity. (**A**) Schematic illustration of how the novel AZ31 magnesium alloy seed strand, in combination with radiotherapy, promotes tumor cell death through mitochondrial dysfunction and DNA damage [104]; (**B**) Mg-Motor-DOX@Gel revealed through RNA-Seq that hydrogen therapy and chemotherapy synergistically promote immune cell infiltration [106]; (**C**) Schematic illustration of P/S/CVA@NPs-AI + laser overcoming ccRCC sunitinib resistance under the cooperation of PT2385, silncARSR and H_2_ [108]; (**D**) In vitro image of P/S/CVA@NPs-AI + laser inhibiting vascular lumen formation [108]. scale bar = 100 μm; (**E**) Quantitative analysis of the M1/M2 ratio after P/S/CVA@NPs-AI + laser treatment [108]; (**F**) Schematic illustration of MnG-mediated hydrogen production regulating tumor metabolism to enhance cGAS-STING activation, thereby promoting immunotherapy [84]; (**G**) Quantitative analysis of IFN-β secreted by mature dendritic cells (DCs) following treatment with MnG and other agents [84]; (**H**) Western blot analysis to determine how MnG regulates the cGAS-STING pathway [84]. NS: not significant; ** *p* < 0.01, *** *p* < 0.001. **** *p* < 0.0001, and the data are presented as the mean ± SD.

Notably, H_2_ can influence cancer cell metabolic reprogramming through pathways such as AMPK/mTOR and PD-1/PD-L1, hereby synergizing with radiotherapy and chemotherapy to treat tumors, while the systemic immune response and T cells activated by H_2_ are highly likely to help maintain antitumor function [103]. Mg and Ca are commonly used as hydrogen-producing metal substrates in combination with radiotherapy and chemotherapy, primarily due to their pH-dependent H_2_ production and ability to neutralize the acidic microenvironment, which, to a certain extent, can reverse tumor resistance and enhance the penetration of chemotherapeutic and radiotherapeutic agents. Hu et al. utilized the pH-responsive H_2_-releasing properties of the AZ231 magnesium alloy to enhance the efficacy of I^125^ radiotherapy [104] (Figure 4A). H_2_ promotes mitochondrial dysfunction, while I^125^ induces DNA damage, and the two act synergistically to accelerate apoptosis. Furthermore, H_2_ not only increases tumor cell sensitivity to chemotherapeutic agents by inhibiting mitochondrial function and blocking ATP synthesis [105], but also modulates the JAK-STAT and NF-κB signaling pathways to enhance immune responses, thereby reversing drug resistance: (1) improving the function and proliferation of CD8^+^ T cells and NK cells [106]; (2) inducing the polarization of M2-type TAMs to M1-type TAMs, thereby enhancing tumor sensitivity to cytotoxic and redox agents [51] (Figure 4B). Liu et al. designed nano-CaH_2_ based on intracellular calcium channel homeostasis. In the OH^−^-regulated low-pH tumor microenvironment, while alleviating T-cell functional suppression, the local increase in Ca^2+^ concentration causes calcium overload in tumor cells, leading to calcium-mediated cell death. Meanwhile, the released H_2_ induces ICD and the release of DAMPs, which are subsequently recognized and presented by mature DCs, thereby activating the immune system [93]. The effective activation of the immune system was demonstrated in both local and distant tumor suppression in mice (Figure 4C). In this synergistic effect, chemotherapy drugs and radiation therapy exert potent ICD induction and cytotoxic effects, while the role of H_2_ is more likely to manifest as the scavenging of excess ROS induced by chemoradiotherapy and the improvement of the tumor microenvironment, thereby protecting effector T cells and enhancing immune cell infiltration, which indirectly prolongs the duration and efficacy of the antitumor immune response.

The rapid proliferation of tumor cells and their unique reliance on glycolysis for energy production readily create a hypoxic tumor microenvironment. Under hypoxic conditions, tumor cells secrete excessive amounts of vascular endothelial growth factor (VEGF) and other factors, leading to abnormal vascular structure [107]. This exacerbates tumor hypoxia and acidification, promotes tumor migration and invasion, and restricts the access of immune cells and drugs. Consequently, abnormal angiogenesis has become a major characteristic of malignant tumors. To overcome the therapeutic resistance associated with abnormal angiogenesis, the researchers employed a synergistic delivery system combining H_2_, the HIF-2α inhibitor PT2385, and siRNA targeting lncARSR (P/S/CVA@NPs-AI) (Figure 4C), and innovatively discovered that under 660 nm NIR irradiation, the H_2_ generated in situ by this composite material could directly reduce vascular endothelial growth factor A (VEGFA) in cancer cells [108]. In synergy with siRNA-ARSR, this effect simultaneously enhances the suppression of VEGFA secretion by M2-type TAMs, while PT2385 inhibits the HIF-2α/VEGFA pathway, and the combined action of these three pathways achieves the inhibition of intratumoral angiogenesis (Figure 4D). However, this synergistic effect does not affect the secretion of pro-angiogenic factors in human umbilical vein endothelial cells. Unlike most cancer treatment strategies that rely on improving the tumor-suppressive microenvironment or inducing oxidative stress, this study uniquely amplifies the therapeutic effects of a gas-and-multidrug combination strategy from an anti-angiogenic perspective and elucidates the mechanism by which H_2_ inhibits tumor angiogenesis (Figure 4E). However, it is worth noting that the molecular mechanism by which H_2_ directly inhibits VEGFA remains unclear. It has not yet been determined whether H_2_ regulates HIF-2α activity via sensor proteins or influences VEGFA transcription by altering intracellular redox status, so further research is needed to explore the direct molecular evidence for this mechanism.

Abnormal vascularization and interstitial fluid accumulation within tumor tissue can lead to increased intratumoral interstitial fluid pressure, severely limiting the penetration of drugs and immune cells into the deep layers of the tumor [107]. This also affects the tissue permeability of solid nanometals, whereas liquid metals stand out due to their fluidity, good biocompatibility, and high conductivity, offering a solution to the problems of poor penetration and limited catalytic efficiency associated with solid metals (Figure 4F). Hao et al. developed a gallium-based liquid metal (Ga_67_In_20.5_Sn_12.5_) catalytic system (LM@M) encapsulated within tumor cell membranes, which exhibits homogeneous targeting, dynamic deformability, and piezoelectric catalytic activity. Under ultrasonic catalysis, LM@M decomposes water to produce H_2_, thereby reducing the interstitial fluid within the tumor and enhancing the penetration efficiency of drugs and immune cells [44]. Simultaneously, LM@M achieves a dual immune activation effect under ultrasonic catalysis: on one hand, the generated H_2_ induces oxidative stress in tumor cells, leading to the release of ICDs and the activation of DCs; on the other hand, the piezoelectric effect of LM@M damages cell nuclei, and the released DNA is recognized by the innate immune receptor cGAS, thereby activating the cGAS-STING signaling pathway to regulate immune effector molecules such as IFN-α and IFN-β, promoting antigen presentation and the infiltration of CD4^+^ and CD8^+^ T cells (Figure 4G,H). The liquid metal catalytic system investigated in this study represents a significant breakthrough in addressing the challenges of catalytic and permeation efficiency in metal catalysis, offering new design concepts for materials that activate hydrogen-mediated immunity. By combining the unique properties of hydrogen therapy with various treatments such as radiation therapy or chemotherapy, and leveraging H_2_’s antioxidant, anti-inflammatory, and anti-apoptotic capabilities to limit the damage caused by radiation and chemotherapy, this approach offers a viable new strategy for reducing the toxic side effects and drug resistance associated with current therapies.

Although the above studies indicate that nanometal hydrogen therapy can activate various immune-related signaling pathways, this serves only as indirect evidence for its role as an adjunctive therapy. Therefore, there remains significant room for exploration regarding direct molecular evidence of how H_2_ molecules regulate immune signaling. While the notion that “iron porphyrins serve as targeted binding sites for H_2_” has been validated in in vitro experiments, whether H_2_ possesses other high-affinity binding targets and which signaling networks it can activate as a single active molecule still requires in-depth investigation using biological techniques such as single-cell sequencing and transcriptomics (Table 3).

## 4. Challenges and Future Prospects of Hydrogen Immunotherapy

### 4.1. Status of Clinical Translation

The clinical translation of metal nanomaterials is hindered by three major challenges: unclear pharmacokinetics, concerns regarding potential toxicity, and difficulties in quality control during large-scale production [113]. As a result, the clinical translation of hydrogen therapy based on metal nanomaterials for cancer treatment has progressed slowly. Currently, most hydrogen therapy projects are concentrated in the experimental animal research phase, while population cohort studies or clinical applications still rely on traditional H_2_ delivery methods such as hydrogen inhalation, hydrogen-rich water, and hydrogen-saline injections; however, their therapeutic effects are limited [114]. We must not overlook the fact that the trend toward clinical applications of metal nanomaterials is becoming increasingly evident, including superparamagnetic iron oxide nanoparticles [115] as MRI contrast agents, as well as platinum-containing complexes such as cisplatin, carboplatin, and oxaliplatin used in cancer treatment [116]. In recent years, an increasing number of metal nanomaterials have entered preclinical trials due to their stable performance and excellent therapeutic and diagnostic effects in animal studies (Table 4).

Although nanometal hydride therapy has demonstrated significant antitumor potential in preclinical studies, its translation into clinical practice still faces multiple obstacles, including unclear pharmacokinetics and biodistribution, concerns about potential toxicity, and difficulties in quality control for large-scale manufacture. These issues are critical to the biosafety and industrial feasibility of the nanoparticles. In terms of pharmacokinetics and biodistribution, the particle size, morphology, surface charge, and protein coat composition of nanomaterials have a profound impact on their circulation half-life, tissue distribution, and tumor-targeting efficiency in vivo. Nanoparticles with cationic surfaces generally exhibit stronger cytotoxicity (2–3 times) [123]. Metal nanoparticles administered intravenously are primarily captured by the mononuclear phagocyte system, such as the liver and spleen; the retention rate in the liver can reach 30–40%, while the actual accumulation efficiency at the tumor site is extremely low, typically less than 5% of the administered dose. Although nanoparticles with a particle size of less than 10 nm possess greater tissue penetration capacity, their renal clearance efficiency is limited, and they exhibit higher genotoxicity and a greater likelihood of off-target effects [124,125]. Currently, most studies have effectively reduced the biotoxicity of nanoparticles to some extent through PEGylation and biodegradable hybrid modifications [126]. Regarding hydrogen release kinetics, there are significant differences in hydrogen production rates and release patterns among different metallic materials. Among them, Mg-, Ca-, Mn-, and Cu-based materials rely on hydrolysis reactions in an acidic microenvironment, and their hydrogen production rates are strongly influenced by local pH. Hydrogen storage materials such as PdH can achieve controlled release triggered by near-infrared light; however, the persistence of hydrogen release and real-time monitoring of in vivo concentrations are difficult to quantify effectively. At the same time, studies have shown that nanoparticles such as Ag, Au, and Pt exhibit dose- and size-dependent effects; the smaller the nanoparticle size and the higher the administered dose (above 20 μg/mL), the greater the reduction in cell viable fraction (by more than 50%) through mechanisms such as disruption of cell membrane integrity and disturbance of K^+^/Ca^2+^ ion homeostasis [127]. However, their long-term metabolic fate in vivo remains unclear. Although degradable metals (such as Mg and Fe) can be gradually metabolized through hydrolysis or enzymatic degradation, the local concentration and clearance rate of their degradation products are difficult to precisely control. Long-term or repeated administration may still lead to the accumulation of metal ions in organs of the mononuclear phagocyte system (such as the liver and spleen). In terms of manufacturing and quality control, the transition of nanomaterial synthesis from laboratory-scale to GMP-compliant industrial production is constrained by differences in production equipment and the difficulty of achieving proportional mixing ratios [128]. This can easily lead to variations in particle size distribution, surface charge, and drug loading between batches, thereby significantly altering their metabolic and pharmacological processes. Currently, only a very small number of nanomedicines (such as Feraheme) have achieved industrial-scale production and received FDA approval [129]. Furthermore, there is a global lack of unified quality standards and definitions for nanomaterials, and the prohibition of TiO_2_ in food in the European Union, France, and other countries may complicate the regulatory review and approval of nanoparticles as pharmaceuticals in certain nations. Regarding long-term safety, the vast majority of current safety assessments are limited to short-term rodent models, making it difficult to fully reflect their potential toxicity, immunological effects, and interspecies differences in pharmacokinetics. Therefore, to advance the clinical translation of nano-metal hydrogen therapy, the aforementioned risk factors must be incorporated as core considerations in material design.

### 4.2. Challenges and Future Prospects

Metal-based nanomaterials, by leveraging their inherent physicochemical properties and specific biological activities (such as nanozymes), can achieve spatially and temporally controlled responsive release in response to external stimuli or the body’s internal environment. As a result, they show significant application potential in areas such as anticancer drug delivery, radiosensitization and chemotherapeutic sensitization, and diagnostic imaging. Hydrogen therapy based on metal nanomaterials combines the Fenton/Fenton-like reactions of these materials with H_2_ as a signaling molecule and its anti-inflammatory and antioxidant properties, demonstrating synergistic therapeutic potential in animal studies [130]. As the entire cancer-immune cycle progresses, metal-based hydrogen therapy exhibits a layered immune activation effect characterized by “inducing antigen release, reversing immunosuppression, enhancing T cell infiltration”. At the same time, by utilizing metal ions and H_2_ to synergistically activate signaling pathways such as cGMP-PKG, PI3K-Akt, NF-κB, JAK-STAT, and cGAS-STING, as well as the lysosomal pathway, this therapy induces cancer cell apoptosis or pyroptosis at the molecular level. However, the aforementioned mechanisms are primarily based on in vitro cellular experiments and observations in animal models and have yet to be systematically validated in clinical trials.

Furthermore, the tumor-specific “immune editing-immune evasion-ITH” dynamic evolutionary process is one of the fundamental causes of tumor diversity, treatment resistance, and variations in clinical efficacy [131]. First, the disordered proliferation of tumors forms a dense structure (termed the ECM), while the complex internal vascular architecture creates high interstitial fluid pressure (IFP). Together, these factors impede the diffusion of drugs from blood vessels into the tumor and limit the effective delivery of NIR light, ultrasound, and thermal stimulation. As a result, the actual power is continuously attenuated during transmission into the tumor, making it difficult to effectively activate responsive metallic materials. Second, most studies use xenograft cancer models in animals, which struggle to mimic the complex TME of in situ cancer formation [132]. This makes it difficult to effectively investigate the pharmacokinetic and immunological effects of hydrogen-producing metallic materials on patients, thereby limiting the clinical translation of nanometallic hydrogen therapy. Finally, there may be a risk of off-target failure with hydrogen-producing metallic materials, and the accumulation of off-target materials in normal organs could pose potential safety risks [133]. Although H_2_ provides some protective effects for normal cells, the non-specific distribution of the nanomaterials themselves and the long-term biological effects of their degradation products still require careful evaluation. Currently, most studies evaluate the biosafety of these materials solely through cell survival rates and histological hematoxylin–eosin (HE) staining of animal model tissues during the treatment period (14–21 days) to determine that the materials possess good biosafety. However, these conclusions lack thorough substantiation from longer-term and systematic safety assessments.

Meanwhile, we have identified numerous properties of H_2_ that offer theoretical potential for its clinical applications: (1) Its extremely small molecular size allows it to cross the blood–brain barrier; (2) the potential biological targets of H_2_ may be related to iron porphyrins [101]; (3) organs and systems highly sensitive to H_2_ include the liver, gastrointestinal tract, brain and central nervous system, and cardiovascular system; and (4) it may have a protective effect on distant organs [134]. It is precisely because of these characteristics that nanometal hydrogen therapy can optimize and validate its clinical translation feasibility from a broader perspective. Precisely because of these characteristics, the clinical translational feasibility of nanometal hydrogen therapy can be preliminarily explored from a broader perspective. Disease models involving organs with high sensitivity to H_2_ should be prioritized. By utilizing a co-culture system of patient-derived tumor organoids and immune cells, the universality of this strategy’s effects and the underlying immune regulatory pathways can be evaluated, providing a reference for material optimization. We will explore “microbial–metal” combined systems [135], leveraging beneficial metabolites (such as short-chain fatty acids) produced when H_2_ is utilized by specific microbial communities, with the aim of amplifying its immunomodulatory effects and synergistically enhancing the potential of anti-tumor immunotherapy [101]. Furthermore, given that potential molecular targets for H_2_ may be iron porphyrins in mitochondria and red blood cells, future material design could adopt a new “targeted delivery-functional enhancement” model. By functionalizing the surfaces of nanomaterials and regulating the electronic structure and crystal configuration of metals, we aim to enhance target-interaction capabilities, thereby improving the antitumor efficacy of hydrogen therapy.

From a long-term perspective, the future development of nanometal hydrogen therapy can focus on the following cutting-edge directions. First, the construction of multi-responsive smart nanoplatforms. This would enable the material’s functional units to be activated by multiple stimuli to produce hydrogen, thereby achieving a cascade response to multiple abnormal signals in the tumor microenvironment (such as acidification and overexpression of specific enzymes), and enhancing the sensitivity and selectivity of the treatment process. Notably, biodegradable metals such as Mg and Ca, which exhibit a relatively low binding energy in response to light or electrical stimulation, offer superior biosafety and controllability, and may represent a cutting-edge direction for clinical translation. Second, the development of biodegradable metallic nanomaterials. To address the inherent drawbacks of precious metals (such as Pt, Pd, and Au) that are difficult to degrade and accumulate over the long term in vivo, researchers in recent years have focused on developing two-dimensional nanosheets based on biodegradable metals such as MoS_2_ and Cu. This allows the materials to be gradually metabolized via enzymatic or hydrolytic pathways during treatment, thereby enhancing the long-term biosafety of the nanomaterials. Third, artificial intelligence (AI)-assisted nanomaterial design and optimization. Deep learning models are used to optimize material synthesis parameters, enhance material performance and therapeutic efficacy, and predict in vivo metabolic or degradation behavior. AI-driven design frameworks can integrate multidimensional data, including the physicochemical properties and immune response effects of validated nanomaterials, thereby establishing a new paradigm for predictable material validation. Fourth, combination therapy with immune checkpoint inhibitors (ICIs) or CAR-T therapy. Existing studies have effectively combined nanometal hydride therapy with anti-PD-1/PD-L1 antibodies or CAR-T cell therapy to synergistically improve the overall response rate of immunotherapy. Fifth, the design of personalized cancer immunotherapy. By integrating large AI models with technologies such as patient-derived tumor organoids, single-cell sequencing, and spatial transcriptomics, the selection of metal types, surface modification strategies, and administration routes can be optimized based on the characteristics of the patient’s specific tumor immune microenvironment.

Furthermore, the issue of H_2_’s molecular targets remains a highly controversial focal point in the field. Hypotheses regarding how H_2_ achieves or enhances cancer treatment primarily fall into four categories: direct scavenging of free radicals, targeting of iron porphyrins, targeting of Rieske iron-sulfide proteins, and the absence of stable binding receptors [136]. While these hypotheses have been supported by some experimental evidence at the cellular and animal levels, it is difficult to specifically distinguish the respective roles played by H_2_ and other metal ions in these processes, and there is a lack of more rigorous evidence, such as kinetic and genetic data. At the same time, we believe that in hydrogen-mediated immunotherapy achieved through hydrogen production by metallic nanoparticles, H_2_ primarily plays a supportive role in improving the tumor’s immunosuppressive microenvironment by clearing excessively accumulated ROS from the microenvironment and protecting and enhancing immune cell infiltration. However, while numerous studies suggest that H_2_ acts as an active signaling molecule involved in immune activation and regulation, the relevant molecular evidence remains relatively limited.

In summary, nano-metal hydrogen therapy represents a shift from traditional non-specific gas therapy strategies toward a precision-controlled therapeutic approach that generates H_2_ in a spatiotemporally responsive manner. Through the development of metal nanomaterials that are targeted, intelligent, synergistic, and controllable, this therapy activates and enhances immune responses to combat tumors. Simultaneously, leveraging the advantages of H_2_ as a signaling molecule to activate innate immune responses and selectively scavenge highly toxic ROS to protect immune cell function, nano-metal hydrogen therapy utilizes metal ions (such as Mn^2+^, Fe^2+^ and Mg^2+^) to trigger key pathways like cGAS-STING, thereby enabling precise intervention during the initiation, activation, and effector phases of the cancer immune cycle. However, these approaches are currently still in the basic research or animal experimentation phase, and their feasibility and efficacy require further experimental evidence to support them. Hydrogen therapy still requires consideration of issues such as potential material toxicity, pharmacokinetic rates, and metal accumulation. This necessitates the interdisciplinary integration of nanomaterials science, synthetic biology, biomedicine, immunology, and clinical medicine to establish an integrated system for evaluation and feedback optimized through “animal models-patient-derived organoid systems-clinical trials”. This approach will address challenges such as material failure caused by tumor heterogeneity, the biosafety of the materials themselves, and the scalability of production. Ultimately, this will advance nano-metal hydrogen therapy from the laboratory to clinical practice, providing a new multimodal treatment paradigm for solid tumors, inflammatory diseases, and wound healing. Only with robust preclinical data and a carefully designed clinical trial can we objectively assess whether hydrogen therapy will ultimately provide new treatment options for indications such as solid tumors, inflammatory diseases, and tissue repair.

The literature search for this review was conducted using the two major databases, Web of Science and PubMed, focusing primarily on research published over the past five years (2021–2026). Search terms included “hydrogen therapy”, “molecular hydrogen”, “H_2_”, “cancer immunotherapy” and “tumor immunity”. After deduplication, screening of titles and abstracts, and full-text evaluation, a total of 119 representative articles were ultimately included.

## Figures and Tables

**Table 2:** Summary of Hydrogen-Producing Metal Nanomaterials for Cancer Therapy via Induction of Immunogenic Cell Death.

Metal Nanomaterials	Cancer Model	Size	Biodegradability and Toxicity	Response Conditions	Active Molecules	Mechanism of Action	Efficacy	Route of Administration	Dosage	Clinical Readiness
MgH_2_-BTO [16]	CT26 cancer model	120 nm	High; no significant toxicity.	Ultrasound stimulation	H_2_, Mg(OH)_2_	MgH_2_ neutralizes the acidic TME, and BTO induces ICD. These two synergistically reverse immunosuppression and enhance T-cell-mediated antitumor responses.	\	Intravenous injections	\	Animal Testing Phase
Nano-CaH_2_ [93]	VX2 orthotopic rabbit liver cancer model	7.9 ± 1.7 nm	High; no significant toxicity.	TME (acidic pH)	Ca^2+^, H_2_, OH^−^	CaH_2_ neutralizes the acidic TME, leading to calcium-overload-mediated cell death.	Eliminate the tumor.	TAE	Rabbit: 0.3 mL 30 μmol	Animal Testing Phase
GNAP [97]	In situ drug-resistant Hepa1-6 liver cancer model	17 nm	High; no significant toxicity.	Light sensitivity (808 nm, 1 W cm^−2^, 10 min)	H_2_, ROS	GNAP neutralizes acid-induced TME, H_2_- and ROS-induced mitochondrial dysfunction, leading to apoptosis and ICD in cancer cells and activating the immune response.	\	Intravenous injections	CNAP: 5 mg kg^−1^; Sorafenib: 10 mg kg^−1^	Animal Testing Phase
Mo_2_C-POM-BiF_3_ (BPM) [43]	4T1 cancer model	40 nm	High; no significant toxicity.	Ultrasound stimulation (0.7 W cm^−2^, 5 min)	H_2_, BiF_3_	BPM inhibits cellular energy metabolism, alleviates tumor hypoxia, and disrupts the redox balance in the TME, thereby inducing tumor cell apoptosis and suppressing proliferation. By increasing the infiltration of DCs, T cells, and M1 macrophages into the TME, it synergistically inhibits tumor growth, thereby triggering a robust antitumor immune response.	\	Intravenous injections	100 μL 2 mg mL^−1^	Animal Testing Phase
BTO/BP-HA [95]	U14 cancer model	150 nm	High; no significant toxicity.	Ultrasound stimulation (1.5 W cm^−2^, 3 min)	H_2_, BP	H_2_ induces mitochondrial damage; BP effectively inhibits the autophagy mechanism activated in tumor cells while simultaneously promoting CTL infiltration, thereby stimulating an antitumor immune response.	\	Intravenous injections	40 μg mL^−1^	Animal Testing Phase
HxMoO_3_@SA@COSs [98]	Acute enteritis model	200 nm	High; no significant toxicity.	Light sensitivity (808 nm, 1 W cm^−2^, 10 min); 12 Gy γ-ray TAI	H_2_	H_2_ reduces radiation-induced tissue damage through its anti-inflammatory and antioxidant effects, promotes the polarization of macrophages toward the anti-inflammatory M2 phenotype, and acts as a synergistic enhancer when combined with NIR.	Survival rate of 80%.	Oral administration	40 mg kg^−1^	Animal Testing Phase
NaGdF_4_:Yb,Tm/g-C_3_N_4_/Cu_3_P@ZIF-8-Folic Acid (UCCZ-FA) [80]	4T1 cancer model	\	High; no significant toxicity.	Light sensitivity (980 nm, 1 W cm^−2^, 5 min)	H_2_, Cu^+^, ROS	Cu^+^ undergoes a redox reaction with GSH, continuously supplying Cu^+^ to ensure the sustained progression of the Fenton reaction and consuming GSH to disrupt the redox homeostasis of cancer cells. Meanwhile, H_2_ and photothermal effects enhance the rate of the Fenton reaction, generating large amounts of ROS to induce apoptosis or ferroptosis in tumor cells. These mechanisms synergistically trigger ICD, releasing DAMPs, thereby activating an antitumor immune response.	Tumors in the UCCZ-FA + laser group were almost completely eliminated.	Intravenous injections	200 μL 1 mg mL^−1^	Animal Testing Phase
MnNi_2_S_3_ NEs [86]	CT26 cancer model	\	High; no significant toxicity.	Electrical stimulation (3.0 V DC, 15 min)	H_2_, ROS	MnNi_2_S_3_ nanocrystals mediate EHT-induced mitochondrial dysfunction and intracellular oxidative stress, reverse the immunosuppressive microenvironment, and trigger pro-inflammatory ICD via pyroptosis, thereby enhancing the infiltration of CD8^+^ T lymphocytes into tumors.	Tumor suppression rate of 100%.	Skin-implanted tumor	0.2 × 0.2 mm^2^	Animal Testing Phase

**Table 3:** Summary of Hydrogen-Producing Metal Nanomaterials for Tumor Therapy via Systemic Immune Activation and Synergistic Enhancement of Chemotherapy and Radiation Therapy.

Metal Nanomaterials	Cancer Model	Size	Biodegradability and Toxicity	Response Conditions	Active Molecules	Mechanism of Action	Efficacy	Route of Administration	Dosage	Clinical Readiness
Nano-CaH_2_ [93]	4T1 and CT26 cancer models	7.9 ± 1.7 nm	High; no significant toxicity.	TME (acidic pH)	Ca^2+^, H_2_, OH^−^	CaH_2_ induces ICD in tumor cells, activating the immune system in synergy with cytotoxic T-lymphocyte-associated antigen 4 (CTLA-4).	Significantly inhibited the growth of in situ tumors and prolonged the survival of mice	Intratumoral injection	Mice: 0.5 μmol nano-CaH_2_+ aCTLA-4 20 μg	Animal Testing Phase
SMND (αPD-1-SF@AB-MSN) [103]	B16-F10 Melanoma	AB-MSN: 240 nm	High; no significant toxicity.	Thermal response (50 °C)	H_2_, αPD-1	H_2_ eliminates excess ROS, works synergistically with αPD-1 to enhance immune cell infiltration, and reduces cancer cell immune evasion.	\	Skin patch	\	Animal Testing Phase
AB/DOX @ HMPDA-PEG [109]	4T1-ADR cancer model	138 nm	High; no significant toxicity.	TME (acidic pH)	H_2_, DOX	H_2_ blocks the mitochondrial respiratory chain and simultaneously sensitizes cells to chemotherapy (DOX).	\	Intravenous injections	100 μL 10 mg kg^−1^	Animal Testing Phase
AMASS (AZ31 magnesium alloy seed strand) [104]	MC38 cancer models; VX2 orthotopic rabbit liver cancer model	\	High; no significant toxicity.	TME (acidic pH)	H_2_, I^125^	H_2_ exacerbates mitochondrial dysfunction and induces apoptosis, and synergizes with I^125^ to enhance radiation-induced DNA damage.	\	Local tumor implantation	\	Animal Testing Phase
L-MgH_2_&TPZ [17]	Rat model of in situ liver cancer	11.07 ± 2.98 nm	High; no significant toxicity.	TME (acidic pH)	H_2_, TPZ, OH^−^, Mg^2+^	H_2_ effectively modulates TME, reverses immunosuppression, promotes drug penetration, interferes with cellular metabolism, enhances the efficacy of chemotherapy, and synergizes with TPZ to kill cancer cells.	Tumors can be eradicated in three weeks.	TAE	50 μL 20 umol L-MgH_2_&TPZ	Animal Testing Phase
Mg-Motor-DOX@Gel [106]	Glioblastoma	23 ± 4 µm	High; no significant toxicity.	Temperature and TME (acidic pH)	H_2_, DOX	H_2_ improves the function and proliferation of CD8^+^ T cells and NK cells, induces the polarization of M2 macrophages into M1 macrophages, activates immune responses, and enhances the efficacy of chemotherapy.	\	Intratumoral injection	10 μL Mg-Motor-DOX@Gel	Animal Testing Phase
Pt(SAc)-MOF@5ASA [51]	CT-26 colorectal cancer	\	High; no significant toxicity.	Light sensitivity (650 nm laser, 100 mW cm^−2^), TME (acidic, high H_2_S and GSH levels)	H_2_, 5-ASA	H_2_-mediated sensitization to anti-inflammatory drugs.	PMA administration significantly reduced tumor volume (90% in the control group, *p* < 0.0001).	Intravenous injections	\	Animal Testing Phase
PdHs-TF [110]	4T1 cancer model	\	High; no significant toxicity.	Light sensitivity (808 nm, 1.5 W cm^−2^, 5 min)	H_2_, TF, OVA	H_2_ inhibits the mitochondrial respiratory system, activates the systemic immune response, and renders cancer cells sensitive to photothermal therapy (PTT).	PdHs-TF effectively inhibits tumor cell metastasis.	Intravenous injections	100 μL 20 mg kg^−1^	Animal Testing Phase
[FeFe]TPP/GEM/FCS NPs [105]	T24 bladder cancer	\	High; no significant toxicity.	Light sensitivity (660 nm, 0.5 W cm^−2^, 10 min)	H_2_, gemcitabine(GEM)	H_2_ induces mitochondrial dysfunction while enhancing the sensitivity to chemotherapy.	Mice survival rates improved significantly, with no deaths observed within 28 days.	Intravesical administration	5.0 mg kg^−1^	Animal Testing Phase
hafnium-bonded hemin (HAHf) [111]	4T1 cancer models	159 nm	High; no significant toxicity.	Ultrasound stimulation (3 W cm^−2^, 10 min)	H_2_, O_2_, ROS, hemin	H_2_ and O_2_ improve the hypoxic environment in the TME, induce ICD to reverse immunosuppression, and enhance the efficacy of radiotherapy.	All treated mice were alive and healthy after 40 days.	Intravenous injections	\	Animal Testing Phase
SnS nanosheets (SSN) [41]	Hepa 1–6-Luc cancer model	160 nm	High; no significant toxicity.	Ultrasound stimulation (3 W cm^−2^, 10% duty cycle, 10 min)	H_2_, LA-PA	H_2_ activates tumor immune responses and alleviates TME-induced immunosuppression.	liver tumor therapy with complete tumor eradication and 100% mice survival	Intravenous injections	100 µL 10 mg kg^−1^	Animal Testing Phase
MnG/Lip-MnG [84]	CT26cancer models; VX2 orthotopic rabbit liver cancer model	\	High; biodegradable; no significant toxicity.	TME (acidic pH)	H_2_, Mn^2+^	H_2_ reverses the immunosuppressive tumor microenvironment, while Mn^2+^ inhibits glycolysis, thereby suppressing cancer cell proliferation.	Lip-MnG inhibits tumor growth and significantly extends survival in mice to 60 days.	Mice: Intratumoral injection; Rabbits: Arterial embolization	Mice: 50 µL 4.0 mg mL^−1^; Rabbits: 0.3 mL 5.0 mg mL^−1^	Animal Testing Phase
P/S/CVA@NPs-AI [108]	Tumors of clear cell renal cell carcinoma resistant to sunitinib (786-O-R cells)	218 ± 2.7 nm	High; no significant toxicity.	Light sensitivity (660 nm)	H_2_, PT2385, lncARSR	H_2_ induces mitochondrial apoptosis and, in synergy with PT2385 (an HIF-2α inhibitor), inhibits angiogenesis, thereby enhancing anticancer efficacy.	The tumor suppression rate reached 95.78%.	Intravenous injections	\	Animal Testing Phase
Au-TiO_2_@ZnS:Cu,Co-A (Au-TiO_2_) @ZnS [112]	MC38 cancer model	210 nm	High; no significant toxicity.	X-ray (1 Gy, 50 kV, 5 min)	H_2_, ROS	H_2_ and Au synergistic radiosensitization for cancer treatment.	The tumor suppression rate reached as high as 90.9% after 15 days.	Intravenous injections	5 mg kg^−1^	Animal Testing Phase
mZnS [15]	4T1 and Hepa1-6 cancer models	90 nm	High; no significant toxicity.	Ultrasound stimulation (1.0 MHz, 3 W cm^−2^, 10% duty cycle, 10 min)	H_2_, O_2_	H_2_ activates the systemic immune response.	After 30 days of treatment, the tumor had virtually disappeared.	Intravenous injections	10 mg kg^−1^	Animal Testing Phase
Ga_67_In_20.5_Sn_12.5_@tumor cytomembranes (LM@M) [44]	U14 cancer model	110 nm	High; no significant toxicity.	Ultrasound stimulation (1.5 W cm^−2^)	H_2_, Tumor stromal pressure	H_2_ disrupts the redox balance and induces tumor cell death, while simultaneously activating the systemic immune response.	\	Intravenous injections	\	Animal Testing Phase

**Table 4:** Summary Table of Clinical Trials on Metal Nanomaterials in Recent Years.

Trial Material	Clinical Trial Registration Number	Core Metal	Clinical Phase	Year	Status	Indications	References
AGulX	NCT02820454 (Completed in 2019) NCT03818386 NCT04899908 NCT04881032	Au	Prospective, randomized open blinded endpoint phase II; Double-blind, randomized phase II; phase I/II	2019; 2021; 2022	Active, not recruiting	Contrast agent; Radiation sensitizers; Glioblastoma	[117,118,119,120]
64Cu-PORPHYSOMES	NCT06977126	Cu	first-in-human (FIH), Phase I	2025	Recruiting	PET/CT Imaging for metastatic/advanced gynecological tumors	\
CNSI-Fe(II)	NCT06048367 (Completed in 2025) NCT07433283 NCT07491783	Fe	Phase Ib/IIa; Multicenter, Open-label Phase II	2024; 2026	Recruiting; Not yet recruiting	Cancer drugs	\
SaNPs	NCT07224464	Fe	Phase I	2025	Recruiting	Cancer drugs	\
Manganese-primed Sintilimab, nab-paclitaxel and platinum chemotherapy	NCT03989336	Mn	Phase II	2019	Completed	Relapsed/Refractory Ovarian Cancer	\
GC4419	NCT02508389	Mn	Phase II	2015	Completed	Oral Mucositis	[121,122]

## Data Availability

No new data were created or analyzed in this study. Data sharing is not applicable to this article.

## Abbreviations

The following abbreviations are used in this manuscript:

H_2_	Hydrogen
ICIs	Immune checkpoint inhibitors
CAR-T	Chimeric antigen receptor T-cell
TME	Tumor microenvironment
CH_4_	Methane
NO	Nitric oxide
CO	Carbon monoxide
H_2_S	Hydrogen sulphide
ROS	Reactive oxygen species
GSH	Glutathione
RNS	Reactive nitrogen species
GSR	Glutathione reductase
ECM	Extracellular matrix
DAMPs	Damage-associated molecular patterns
ICD	Immunogenic cell death
TAMs	Tumor-associated macrophages
Tregs	Regulatory T cells
LM	Liquid metals
·OH	Hydroxyl radicals
EDT	Electrodynamic therapy
DCs	Dendritic cells
AMF	Alternating magnetic field
CAFs	Cancer-associated fibroblasts
TAAs	Tumor-associated antigens
HER	Hydrogen evolution reaction
ETH	Electric field thermotherapy
IMM	Inner mitochondrial membrane
TAE	Transarterial embolization
SDT	Sonodynamic therapy
BP	Black phosphorus
TIP	Tumor interstitial pressure
POM	Polyoxometalate
CRT	Calreticulin
VEGF	Vascular endothelial growth factor
IFP	Interstitial fluid pressure
NIR	Near-infrared
